# Identification and validation of RNA-binding protein-associated biomarkers in esophageal cancer

**DOI:** 10.1186/s41065-026-00726-2

**Published:** 2026-08-07

**Authors:** Jie Gao, Rizhu Li, Xiangsheng Wu, Xueping Feng

**Affiliations:** 1 School of Basic Medical Sciences, Youjiang Medical University for Nationalities, Baise, Guangxi 533000 PR China; 2 School of Clinical Medicine, Youjiang Medical University for Nationalities, Baise, Guangxi 533000 PR China

**Keywords:** Esophageal cancer, RNA-binding proteins, Biomarker, GSEA, Immunoinfiltration

## Abstract

**Background:**

Esophageal cancer (ESCA) is highly prevalent globally, but the role of RNA-binding proteins (RBPs) in it remains unclear.

**Methods:**

ESCA datasets were analyzed to identify differentially expressed genes (DEGs). Candidates were derived by intersecting DEGs with RBP-related genes and validated via machine learning, expression analysis, and receiver operating characteristic (ROC) curves. Prognostic value, functional enrichment, immune microenvironment, regulatory networks, drug prediction, and Eca-109 cell validation were assessed.

**Results:**

Four biomarkers (CDC20, COL7A1, DNMT3B, UBE2T) were confirmed. Low UBE2T/DNMT3B correlated with better survival. In addition, CDC20/UBE2T were linked to DNA replication; COL7A1/DNMT3B to olfactory transduction. Immune analysis showed 15 cell types differed significantly, with most chemokines negatively correlating with biomarkers.

**Conclusions:**

COL7A1, DNMT3B, and UBE2T were associated with transcription factor TET1. The four biomarkers corresponded to 79, 26, 67, and 23 targeted drugs, respectively, and all were highly expressed in Eca-109 cells, advancing insights into RBP-related mechanisms in ESCA.

**Supplementary Information:**

The online version contains supplementary material available at https://doi.org/10.1186/s41065-026-00726-2.

## Background

Esophageal cancer (ESCA), encompassing two primary histological subtypes—esophageal squamous cell carcinoma (ESCC) and esophageal adenocarcinoma (EAC)—is a highly malignant tumour of the gastrointestinal system [1]. ESCA is characterized by high incidence, strong invasiveness, elevated mortality, and poor prognosis. In terms of incidence, it ranks seventh among malignant tumors and sixth globally in terms of cancer-related deaths [2]. Moreover, China reported 301,000 related deaths and 324,000 new ESCA cases in 2020, accounting for 53.70% and 55.35% of the worldwide burden, respectively, according to the World Health Organisation (WHO) [3]. Dysphagia, weight loss and other typical symptoms generally manifest in the intermediate and advanced stages of esophageal cancer. The overall cure rate of combined surgical resection, radiotherapy and chemotherapy is less than 10%. Key challenges including sorafenib resistance, immunosuppressive tumor microenvironment, abnormal glycolysis and excessive cancer stem cell activity greatly impair therapeutic outcomes. Hence, in-depth research is required to clarify the pathogenic mechanisms of esophageal cancer and discover valid diagnostic biomarkers and therapeutic targets [4–7]. Cigarette smoking represents a major environmental risk factor for both ESCC and EAC. It triggers intratumoral inflammatory responses through neutrophil activation and remodels the interaction between immune cells and tumor epithelial cells, thereby accelerating malignant progression [8]. 

Existing research has confirmed that esophageal cancer is regulated by a multi-tiered oncogenic network consisting of transcription factors, long non-coding RNAs (lncRNAs), tumor-specific immune subpopulations and metabolic reprogramming pathways [9]. First, the transcription factor E2F1 drives sorafenib resistance in esophageal cancer cells by transcriptionally repressing tumor-suppressive miR-29c-3p and relieving its inhibitory effect on COL11A1, thereby boosting cell proliferation, cell cycle progression and anti-apoptotic capacity [6]. Second, oncogenic lncRNAs such as DUXAP8 and BBOX1-AS1 facilitate esophageal cancer progression via the competing endogenous RNA (ceRNA) mechanism. DUXAP8 sponges tumor-suppressive microRNAs including miR-29c-3p and recruits epigenetic modifiers EZH2 and LSD1 to silence anti-oncogenic genes, and its overexpression is strongly correlated with tumor advancement and unfavorable prognosis [10]. BBOX1-AS1 is significantly upregulated in ESCC; it targets miR-506–5 A to stabilize EIF5A and subsequently activates the Hedgehog signaling pathway, promoting esophageal cancer cell proliferation and stem cell renewal [11]. Third, transcription factor YY2 serves as a poor prognostic biomarker for esophageal cancer. Its high expression triggers the infiltration of M2 macrophages and regulatory T cells (Tregs), remodels an immunosuppressive microenvironment, and regulates tumor glycolysis. A YY2-centered glycolysis risk signature can effectively stratify the survival outcomes of EAC patients [7]. In addition, single-cell sequencing analyses reveal prominent immune heterogeneity within the esophageal tumor microenvironment. HSPA6⁺ macrophages establish an immunosuppressive niche through ligand-receptor crosstalk with surrounding cells, which negatively impacts patient prognosis [12]. Tobacco exposure activates specific neutrophil subsets, which trigger inflammation and epithelial-mesenchymal transition (EMT) via NAMPT and ICAM1 ligand-receptor axes to accelerate tumor invasion and metastasis [8]. Taken together, transcription factors, lncRNAs, immune cells, glycolysis and Hedgehog signaling coordinately modulate sorafenib resistance, immune evasion, metabolic reprogramming and cancer stem cell traits in esophageal cancer. Nevertheless, the upstream post-transcriptional regulatory machinery that orchestrates this multi-layered oncogenic network remains largely uncharacterized.

RNA-binding proteins (RBPs) constitute a large family of proteins that interact with RNA to regulate multiple RNA metabolic processes, including RNA synthesis, alternative splicing, chemical modification, translocation, and translation, thereby influencing diverse cellular biological functions [13]. RBPs play pivotal roles in signal transduction, particularly through their regulation of microRNAs (miRNAs) and messenger RNAs (mRNAs). Specifically, RBPs modulate miRNA biogenesis by directly binding to primary or precursor miRNAs, or by interacting with miRNA processing machinery; meanwhile, they participate throughout the entire mRNA lifecycle, exerting critical post-transcriptional regulatory functions [14]. Dysregulation of RBPs is closely associated with cancer: aberrant RBP expression can serve as a prognostic indicator and drive tumor initiation and progression by disrupting post-transcriptional control [13, 14]. Notably, RBPs may also modulate tumor cell sensitivity to chemotherapy by binding to the 3’-untranslated region (3’-UTR) of specific mRNAs to promote or suppress translation [15]. Evidence across multiple malignancies supports these roles—for instance, RBP-related genes negatively affect prognosis in lung squamous cell carcinoma (LUSC) via PHF5A-TOMM22-mediated oxidative phosphorylation and TLR3-CTSO-associated inflammatory signaling; [16]POP7 downregulation correlates with worse survival and an immunosuppressive microenvironment in clear cell renal cell carcinoma (ccRCC); [17]and elevated SAM68 expression predicts poor outcomes in bladder and cervical cancers [18, 19], while MSI1 and MSI2 are similarly unfavorable prognostic factors in hepatocellular carcinoma and cervical cancer, respectively [20, 21]. 

In the context of ESCA specifically, accumulating evidence implicates RBPs in disease progression. LincRNA-P21 negatively regulates CDH5 expression via RBP YTHDC1 in ESCC; its loss promotes cell proliferation, migration, and extracellular matrix (ECM) remodeling through CDH5 upregulation [22]. CircRNA circ-FIRRE stabilizes GLI2 mRNA through interaction with RBP HNRNPC, upregulating MYC, CCNE1, and CCNE2 and activating the Hedgehog pathway to drive tumor progression, highlighting the circ-FIRRE/HNRNPC-GLI2 axis as a potential biomarker and therapeutic target [23]. Furthermore, RBP dysregulation facilitates ESCC onset and progression by modulating spliceosomes, RNA transport, and ribosome biogenesis, with genes such as PABPC1 closely associated with clinical stage and survival [24]. Despite these advances, and although targeted therapies against RBPs—including small-molecule drugs, inhibitors, small peptides, and antisense oligonucleotides—have entered clinical trials, the specific molecular mechanisms of RBPs in ESCA remain incompletely understood, representing a critical knowledge gap that warrants systematic investigation.

In this study, we aimed to systematically identify and validate RBP-associated biomarkers in ESCA by integrating transcriptome sequencing data from public databases with differential expression analysis and machine learning approaches. Pathway enrichment, expression correlation, and functional similarity analyses were subsequently performed to characterize the biological roles of the identified biomarkers. A regulatory network was further constructed to reveal potential molecular interactions underlying ESCA progression. Genetic mutation profiles, immune microenvironment characterization, and drug target prediction were also conducted to comprehensively assess the clinical relevance of these biomarkers. Collectively, this study provides new mechanistic insights into RBP-mediated regulation in ESCA and offers a foundation for the discovery of novel therapeutic targets and the development of more effective treatment strategies.

## Methods

### Source of data

The TCGA-ESCA dataset was sourced from the University of California Santa Cruz Genomics Institute Database (UCSC) (https://xenabrowser.net/datapages/). This dataset was chosen as the training set, comprising tumor tissue samples from 161 ESCA patients and 11 samples of para-cancer tissue (controls) [25]. Additionally, the GSE53625 dataset (GPL18109 platform) was acquired from the Gene Expression Omnibus (GEO) database (https://www.ncbi.nlm.nih.gov), which included 179 tumor tissue samples from ESCA and 179 normal para-cancer tissue samples. Furthermore, a total of 1,970 RNA-binding proteins-related genes (RBPRGs) were sourced from the RNA-binding proteins target database (RBPTD) (http://rbptd.com/) [26]. 

### Differential expression analysis

“DESeq2” (v 3.46.0) [27] was used in the training set to filter DEGs between ESCA and control samples (p.adj < 0.05 and |log_2_ Fold Change (FC)| > 2). It should be noted that to focus on transcripts with strong biological effects that could drive malignant phenotypes of ESCA and eliminate meaningless subtle transcriptional fluctuations, a relatively stringent screening threshold of |log₂FC| > 2 was set in this study [28, 29]. This criterion helped screen genes with substantial expression differences, filter background noise caused by tumor heterogeneity, and ensure the reliability of differentially expressed gene screening results. Next, “ggplot2” (v 3.4.1) [30] and “ComplexHeatmap” (v 2.14.0) [31] were used to visualise the DEGs, respectively. Interestingly, the heat map only showed the top 10 DEGs that were up- and down-regulated.

### Identification and analysis of candidate genes

The “ggvenn” package (v 0.1.9) was utilized to visualize the results of candidate genes [32], derived from the intersection of DEGs and RBPRGs. Furthermore, enrichment studies were conducted using the “clusterProfiler” package (v 4.7.1.3), Gene Ontology (GO), and Kyoto Encyclopaedia of Genes and Genomes (KEGG) to investigate the functional functions of these candidate genes in ESCA (*p* < 0.05). The “GOplot” software (v 1.0.2) was used to show the GO keywords, which included molecular functions (MF), cellular components (CC), and biological processes (BP). In addition, the STRING database (http://www.string-db.org/) was used to construct the interaction network with a unified interaction confidence threshold set at ≥ 0.40. Under this screening criterion, some genes failed to form valid interactions with other nodes in the network and were defined as isolated nodes. To focus on core interaction modules and reduce interference from redundant information, these isolated nodes were removed. Cytoscape (v 3.8.2) was subsequently applied to construct the protein-protein interaction (PPI) network for analyzing the associations among candidate genes at the protein level [33]. 

### Biomarker identification

Based on the candidate genes, two machine learning methods were used to identify feature genes: least absolute shrinkage and selection operator (LASSO) and support vector machine-recursive feature elimination (SVM-RFE). Specifically, the “glmnet” package (v 4.1.4) was used to conduct 10-fold cross-validation for LASSO analysis, and the findings were validated at the lowest lambda value. The “caret” package (v 6.0.93) [34] was used to perform SVM-RFE analysis on candidate genes, and an SVM classifier was constructed with the radial basis kernel function (method = “svmRadial”). To avoid model overfitting and obtain unbiased estimation of generalization performance, 5-fold cross-validation (method = “cv”, number = 5) was adopted for resampling evaluation of feature subsets. Model performance was primarily assessed using Accuracy as the evaluation metric. Subsequently, the final set of feature genes was determined by taking the intersection of genes screened by SVM-RFE and LASSO. Expression differences of these feature genes between ESCA and control groups were validated via Wilcoxon tests in the TCGA-ESCA and GSE53625 datasets. Genes with significant intergroup differences (*p* < 0.05) and consistent expression trends across both datasets were designated as candidate key genes. In both datasets, receiver operating characteristic (ROC) curves were then produced for these potential important genes using the “pROC” program (v 1.18.0) [35]. ESCA biomarkers were defined as genes with an area under the curve (AUC) greater than 0.7.

### Prognostic value assessment of the biomarkers

To evaluate the prognostic value of biomarkers, we performed Kaplan-Meier (K-M) analysis. First, ESCA patients from the TCGA-ESCA dataset with complete survival information were divided into groups with high and low expression levels for each biomarker based on optimal cut-off values. The overall survival (OS) and disease-specific survival (DSS) of the two expression groups were then evaluated using K-M curves (*p* < 0.05).

### Functional annotation of biomarkers

To gain further understanding of the biomarkers, the spearman correlation analysis was performed in the expression of biomarkers in TCGA-ESCA dataset. Additionally, the package “RCircos” (v 1.2.2) was used to map biomarkers at corresponding positions on chromosomes [36]. Functional similarity (functional similarity score > 0.5) between biomarkers was analyzed with the help of “GOSemSim” (v 2.24.0) [37]. Gene Set Enrichment Analysis (GSEA) was performed using the GSEA() function implemented in the “clusterProfiler” package. The reference gene set c2.cp.v2025.1.Hs.symbols.gmt from the MSigDB database, which contained canonical pathways including KEGG and Reactome, was adopted. Gene-set permutation was used for significance assessment, with the number of permutations (nPerm) set to 1,000 [38]. Weighted enrichment statistics were applied to calculate enrichment scores to emphasize genes with substantial expression changes. Empirical phenotype permutation was employed to estimate the null distribution of enrichment scores, and normalized enrichment scores (NES) and nominal P values were calculated accordingly (|NES| > 1; *P* < 0.05). We further explored the genetic mutations of biomarkers based on cBioPortal (https://www.cbioportal.org) database of biomarkers for mutation analysis.

### Immune infiltration analysis

Correlations between biomarkers, chemokines, immunoinhibitors and immunostimulators and the tumor immune microenvironment were retrieved from the TISIDB database (http://cis.hku.hk/TISIDB). For TCGA-ESCA esophageal carcinoma samples, the ssGSEA algorithm implemented in the R package GSVA was applied to quantify the infiltration levels of 28 types of immune cells. The standardized gene signatures applied to quantify the infiltration scores of 28 immune cell subtypes were derived from a published immunogenomic study [39]. Differential immune cells were obtained using the Wilcoxon method to compare differences in 28 immune infiltrating cells (*p* < 0.05). Subsequently, the “psych” package was utilized to examine the Spearman correlation between biomarkers comprising diverse immune-infiltrating cells and cellular regulators (|r| > 0.3, *p* < 0.05) [40].

### Regulatory network and drug prediction analyses of biomarkers

The TarBase database (https://dianalab.e-ce.uth.gr/tarbasev9) and NetworkAnalyst database (https://www.networkanalyst.ca/NetworkAnalyst/) [36] were used to estimate the upstream miRNAs and putative transcription factors (TFs) that correlate to biomarkers, respectively [41]. The intersection of the two databases’ predictions was then used to determine the miRNAs. After that, “Cytoscape” was used to map the TF-mRNA and miRNA-mRNA networks. Potential drugs were predicted based on biomarkers using the DSigDB (https://dsigdb.tanlab.org/DSigDBv1.0/), and the these potential drugs were then selected to constructe a drug-biomarker network by “Cytoscape”.

### Cell culture

ESCA cell line Eca-109 was purchased from sunncell biotechnology (China, No. SNL-511), and EC cell line KYSE-410 was purchased from sunncell biotechnology (China, No. SNL-346), and were cultured in medium supplemented with 10% foetal bovine serum (FBS, Gemini BioProducts, Woodland, CA, USA) and DMEM (Thermo Fisher Scientific, CN) medium. Every cell line was kept at 37 °C in an incubator with 5% CO_2_, and the media was replaced every 48 h until the cells achieved the right confluence, the cell generation used was generation 5.

### Quantitative reverse transcription-polymerase chain reaction (RT-qPCR)

RNA was extracted from the human ESCA cell lines Eca-109 and KYSE-410 using TRIzol (Invitrogen, Waltham, MA, USA). cDNA was synthesised and then analysed for quantitative RNA expression by using the SYBR Green PCR Master Mix in a real-time PCR system (Takara Bio, item number RR420A, China). PCR system for quantitative expression analysis of RNA, using β-actin as an internal reference. Each sample underwent three duplicate studies, and Graphpad Prism 10 software was used to analyse and analyse the RT-qPCR data, and the 2^−∆∆Ct^ approach was used to assess the gene expression level. The primer sequences (Kedai Biotechnology, Nanning, China) involved in this study were as follows:

**Table:** 

Gene	Forward Primer (5’→3’)	Reverse Primer (5’→3’)
CDC20	CAAGGAGAACCAGCCTGAAA	GGATCTTGGCTTCCTCTACATC
COL7A1	TTCTTCGCCGTGGATGATG	CTTTGGACAATACACTGGGCAG
DNMT3B	AGGGAAGACTCGATCCTCGTC	GTGTGTAGCTTAGCAGACTGG
UBE2T	ATCCCTCAACATCGCAACTGT	CAGCCTCTGGTAGATTATCAAGC
β-actin	AGCGAGCATCCCCCAAAGTT	GGGCACGAAGGCTCATCATT.

### Analytical statistics

R was used to conduct the statistical analysis (version 4.2.2). Statistical significance was determined by calculating adjusted p-values below 0.05 or P-values less than 0.05.

## Results

### Identification of candidate genes

**Fig. 1:**
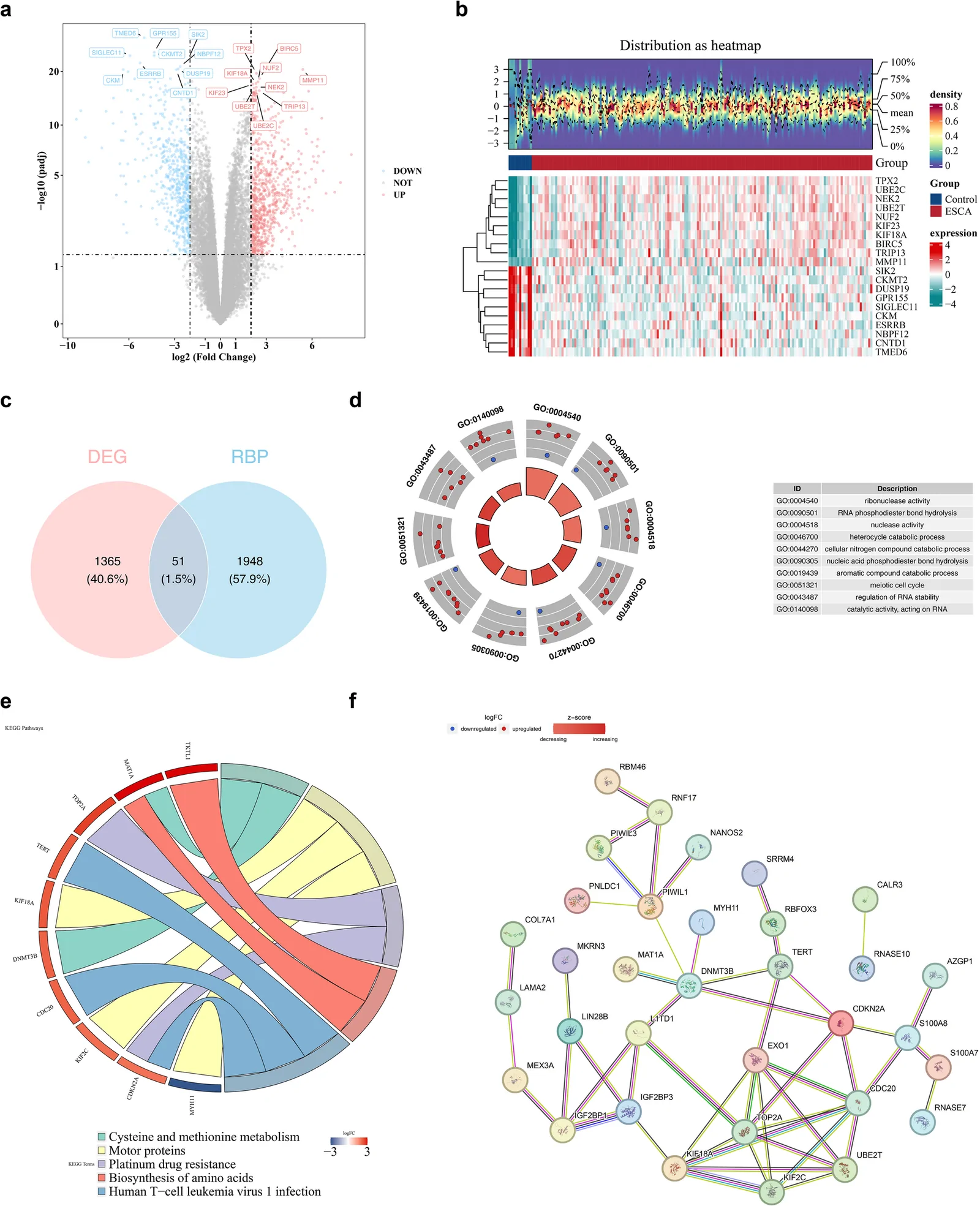
Identification and functional characterization of esophageal cancer and RNA-binding protein-related genes (RBPRGs). **a** Volcano plot of differentially expressed genes (DEGs). Blue represented down-regulated genes, red represented up-regulated genes, and gray represented genes with no significant differential expression; the higher the density and expression, the redder the color; (**b**) Heatmap of DEGs. Blue represents the control group and red represents the esophageal cancer (ESCA) group. The higher the density and expression, the redder the color; (**c**) Venn diagram of DEGs and RBPRGs, illustrating the overlap between RNA-binding protein-related genes and esophageal cancer differentially expressed genes; (**d**) Candidate gene Gene Ontology (GO) enrichment analysis, which enriched 185 BPs (biological processes), 26 CCs (cellular components), and 35 MFs (molecular functions); (**e**) Candidate gene Kyoto Encyclopedia of Genes and Genomes (KEGG) enrichment analysis, showing the top 5 pathways with the most significant enrichment results; (**f**) Candidate gene PPI network, which included 33 nodes with 50 edges

Among the 1,416 DEGs obtained between ESCA and control group in TCGA-ESCA dataset, a total of 600 genes were down-regulated and 816 genes were up-regulated in ESCA (Fig. 1a, b). Subsequently, the intersection of 1,416 DEGs and 1,970 RBPRGs yielded 51 candidate genes (Fig. 1c). GO enrichment analysis was presented in three parts to explore the potential functions of candidate genes (Fig. 1d). For BP, candidate genes were significantly associated with RNA phosphodiester bond hydrolysis, heterocyclic catabolism, cellular nitrogen compound catabolism, nucleic acid phosphodiester bond hydrolysis, and aromatic compound catabolism. Two of the top five BP terms were related to various Nucleic acid phosphodiester bond hydrolytic metabolism. The CC were mainly enriched for the constituents of Cytoplasmic ribonucleoprotein granules, ribonucleosomes, P-bodies, P-particles, poles. The MF indicated that candidate genes were mostly involved in Ribonuclease activity, nuclease activity, endonuclease activity etc. Furthermore, KEGG enrichment analysis revealed the metabolic and signal transduction pathways in cysteine and methionine metabolism, motor proteins, platinum resistance, amino acid biosynthesis, MicroRNAs in cancer, and glycosaminoglycan degradation etc. (Fig. 1e). Moreover, after removing 18 outlier genes, the PPI network showed a close connection among the remained 33 genes (such as CDC20-EXO1, TOP2A-KIF18A, and EXO1-KIF2C ) (Fig. 1f). All enrichment and PPI results represented bioinformatic correlation predictions without cellular functional validation.

### CDC20, COL7A1, DNMT3B, and UBE2T were identified as biomarkers

**Fig. 2:**
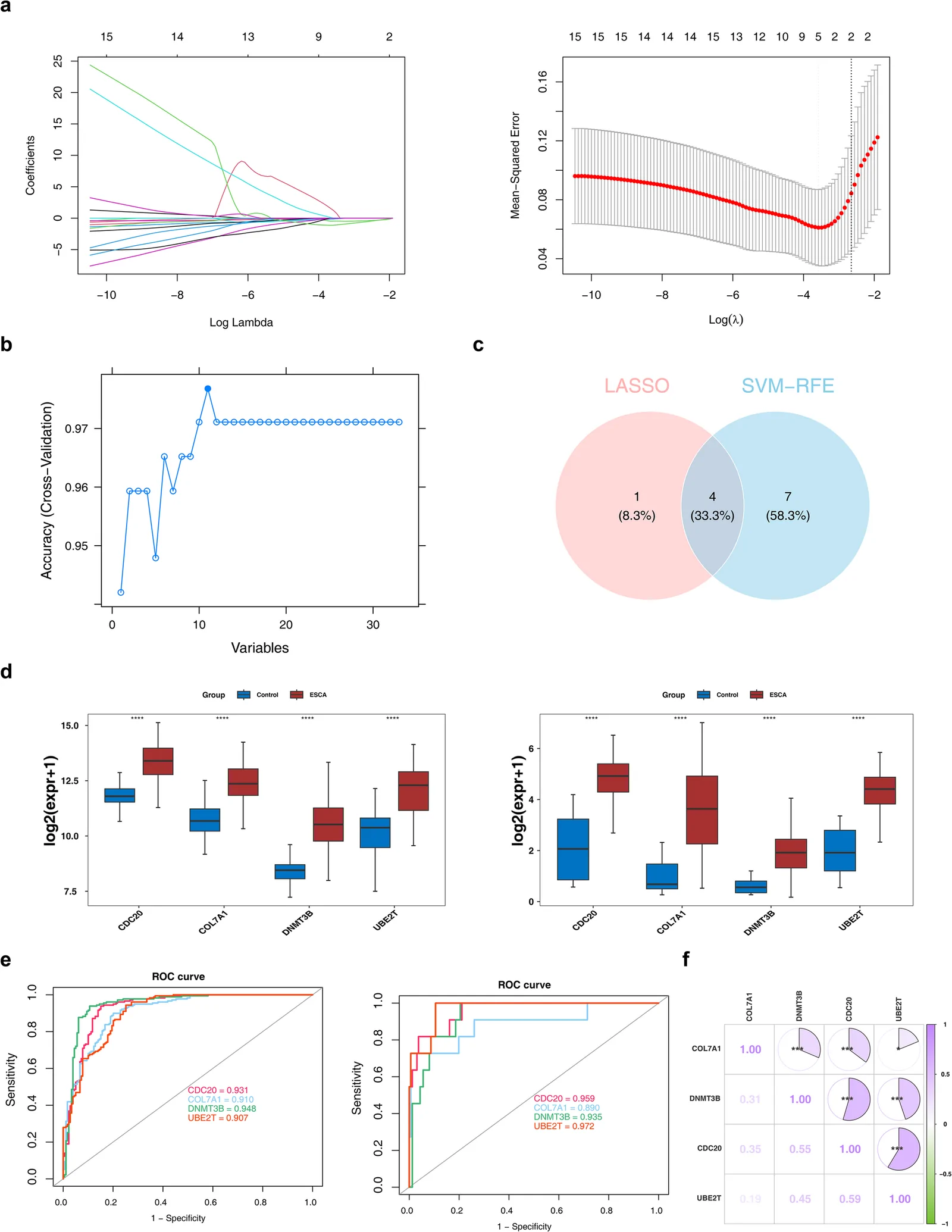
Machine learning screening of key genes. **a** Least Absolute Shrinkage and Selection Operator (LASSO) method for screening key genes. The left figure shows the spectrum of LASSO coefficients. The horizontal coordinate is the logarithm of lambdas and the vertical coordinate is the variable coefficients, and the right figure shows the ten-fold cross-validation of adjusted parameters in LASSO analysis. The horizontal coordinate is the logarithm of lambdas and the vertical coordinate is the model error; (**b**) support vector machine-recursive feature elimination (SVM-RFE) method for screening key genes. The horizontal coordinate is the number of characterized genes and the vertical coordinate is the model prediction accuracy; (**c**) Identification of key genes. Illustration of the overlap between the key genes screened by the LASSO method and the SVM-RFE method; (**d**) Validation of the expression of key genes in the dataset The right panel shows the training set TCGA-ESCA, and the left panel shows the validation set GSE53625, box-and-line plots are genes in the horizontal coordinates; expression in the vertical coordinates, disease samples in red, and control samples in blue; and asterisks are significance: ,*p < 0.05, **p < 0.01, ***p < 0.001, ****p < 0.0001; (**e**) ROC validation of key genes. The right panel shows the training set TCGA-ESCA, and the left panel shows the validation set GSE53625; (**f**) key genes correlation heatmap. Horizontal and vertical coordinates are biomarkers, numbers indicate correlations, and asterisks indicate significance: ,*p < 0.05, **p < 0.01, ***p < 0.001

The 4 feature genes (CDC20, COL7A1, DNMT3B, UBE2T) were identified by overlapping the 5 genes from LASSO and 11 genes from SVM-RFE (Fig. 2a-c). In addition, the expression of the 4 feature genes were found to be consistent and significantly different in TCGA-ESCA and GSE53625 datasets (*p* < 0.05) (Fig. 2d). Moreover, ROC analysis demonstrated that CDC20, COL7A1, DNMT3B, and UBE2T had AUC values exceeding 0.7 in both datasets, indicating their robust discriminatory potential between ESCA and control samples (Fig. 2e). Therefore, these 4 genes were designated as biomarkers. Among them, CDC20 and DNMT3B possessed a high correlation based on Spearman method (*r* = 0.55, *p* < 0.05) (Fig. 2f). Transcript-level expression correlation only reflected statistical trends and could not confirm causal interactions between genes.

### DNMT3B and UBE2T demonstrated outstanding prognostic value

**Fig. 3:**
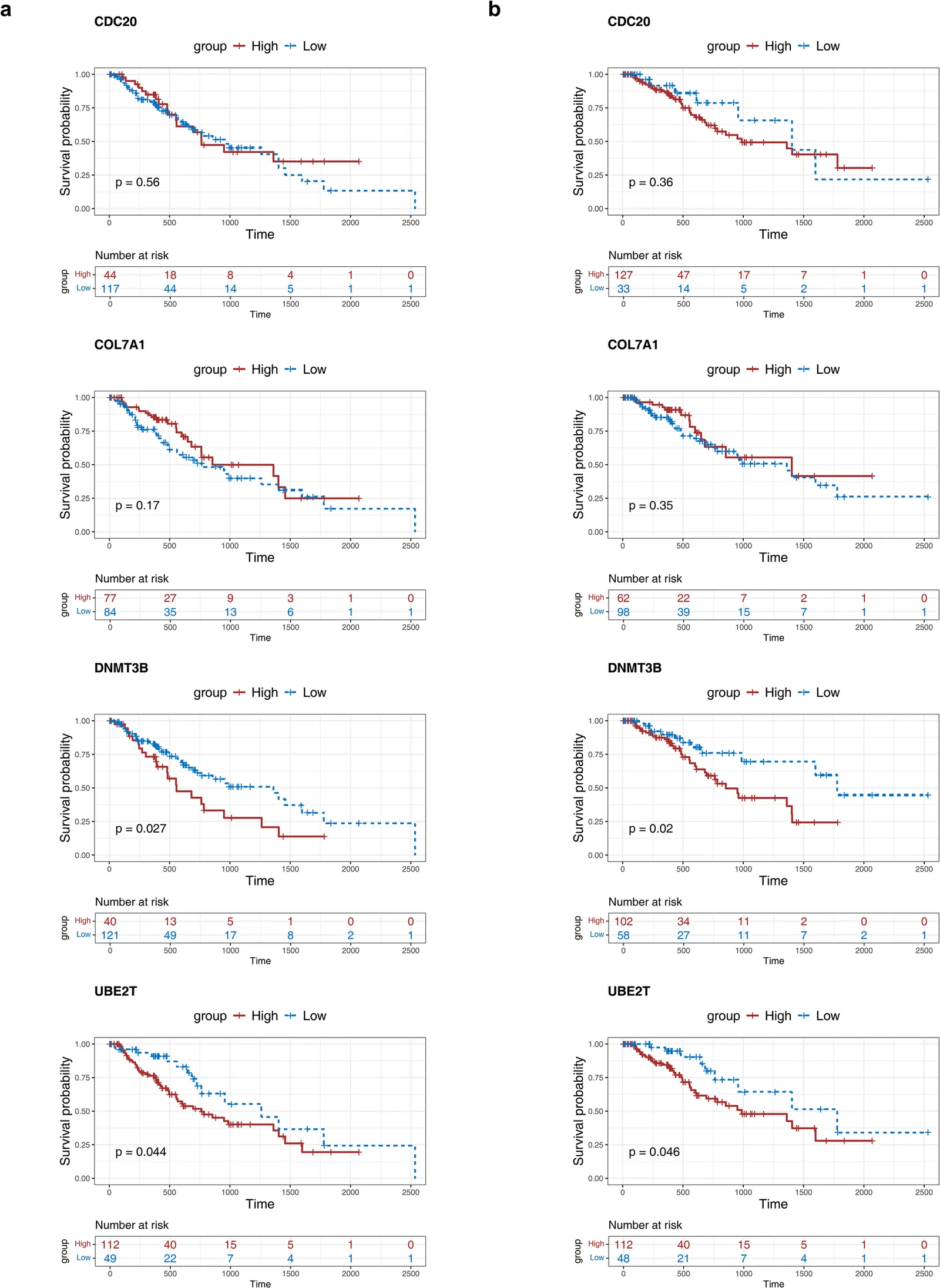
Analysis of the prognostic value of biomarkers. **a** Survival curve of high and low biomarker expression groups in Overall Survival (OS); (**b**) Survival curve of high and low biomarker expression groups in Disease-Specific Survival (DSS)

In the K-M curves predicting OS for ESCA patients, those with low expression patients of DNMT3B (*p* = 0.027) and UBE2T (*p* = 0.044) exhibited significantly higher survival probabilities and greater survival durations in contrast to the group with high expression (Fig. 3a). Similarly, K-M curves predicting DSS revealed that individuals with low levels of UBE2T (*p* = 0.046) and DNMT3B (*p* = 0.02) expression also had significantly higher survival probabilities and extended survival times compared to their high-expression counterparts (Fig. 3b). These findings underscored the potential of DNMT3B and UBE2T as valuable prognostic biomarkers for ESCA, offering insights into patient outcomes. Survival differences represented statistical correlations rather than proven causal regulatory effects on patient prognosis.

### Exploring biomarkers by chromosomal localization, functional analysis, and genetic variation analyses

**Fig. 4:**
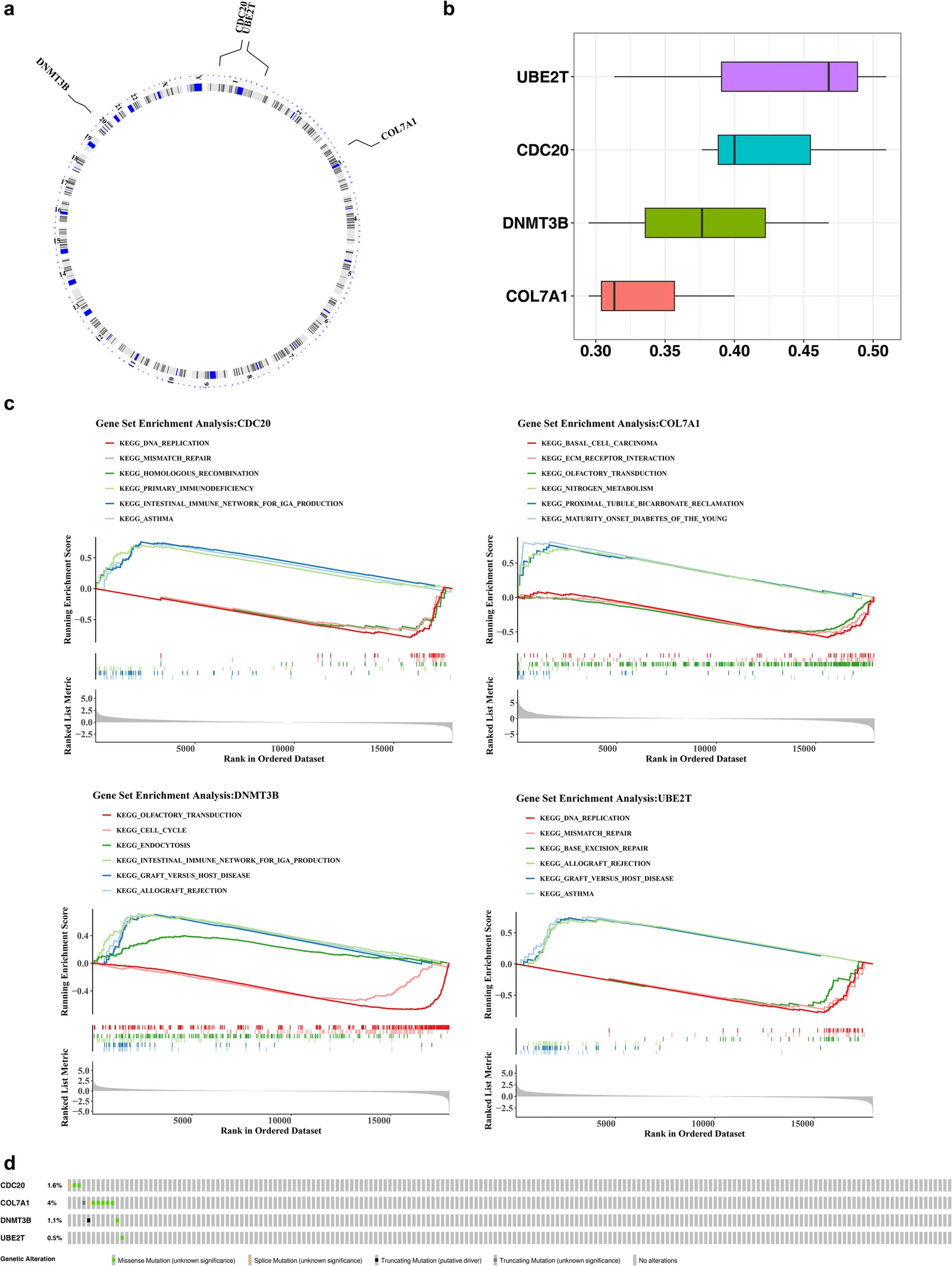
Biomarker correlation analysis. **a** Biomarker chromosomal localization analysis; (**b**) Biomarker functional similarity analysis; (**c**) Biomarker GSEA enrichment analysis; (**d**) Biomarker mutation frequency

The visualization of chromosome positions in ESCA and normal controls, with the CDC20 and UBE2T were located on chromosome 1, COL7A1 was located on chromosome 3, and DNMT3B was located on chromosome 20 (Fig. 4a). The results of the functional similarity demonstrated that the functional similarity between UBE2T and other biomarkers was highest (Fig. 4b). In addition, GSEA showed that UBE2T and CDC20 were mainly enriched in DNA replication, mismatch repair, and homologous recombination pathways, DNMT3B was primarily associated with cell cycle regulation, and COL7A1 was enriched in ECM-receptor interaction and tumor invasion-related pathways, implying potential correlative involvement in genome maintenance, cell proliferation and stromal microenvironment remodeling (Fig. 4c). The genetic mutations in CDC20, COL7A1, DNMT3B, and UBE2T were further explored, and the results displayed that the mutation rates of CDC20, COL7A1, DNMT3B, and UBE2T were 1.6%, 4%, 1.1%, and 1.1%, respectively (Fig. 4d). Collectively, GSEA and functional similarity outputs were predictive pathway correlations lacking direct cellular functional verification.

### Analysis of 28 types of immune cell infiltration

**Fig. 5:**
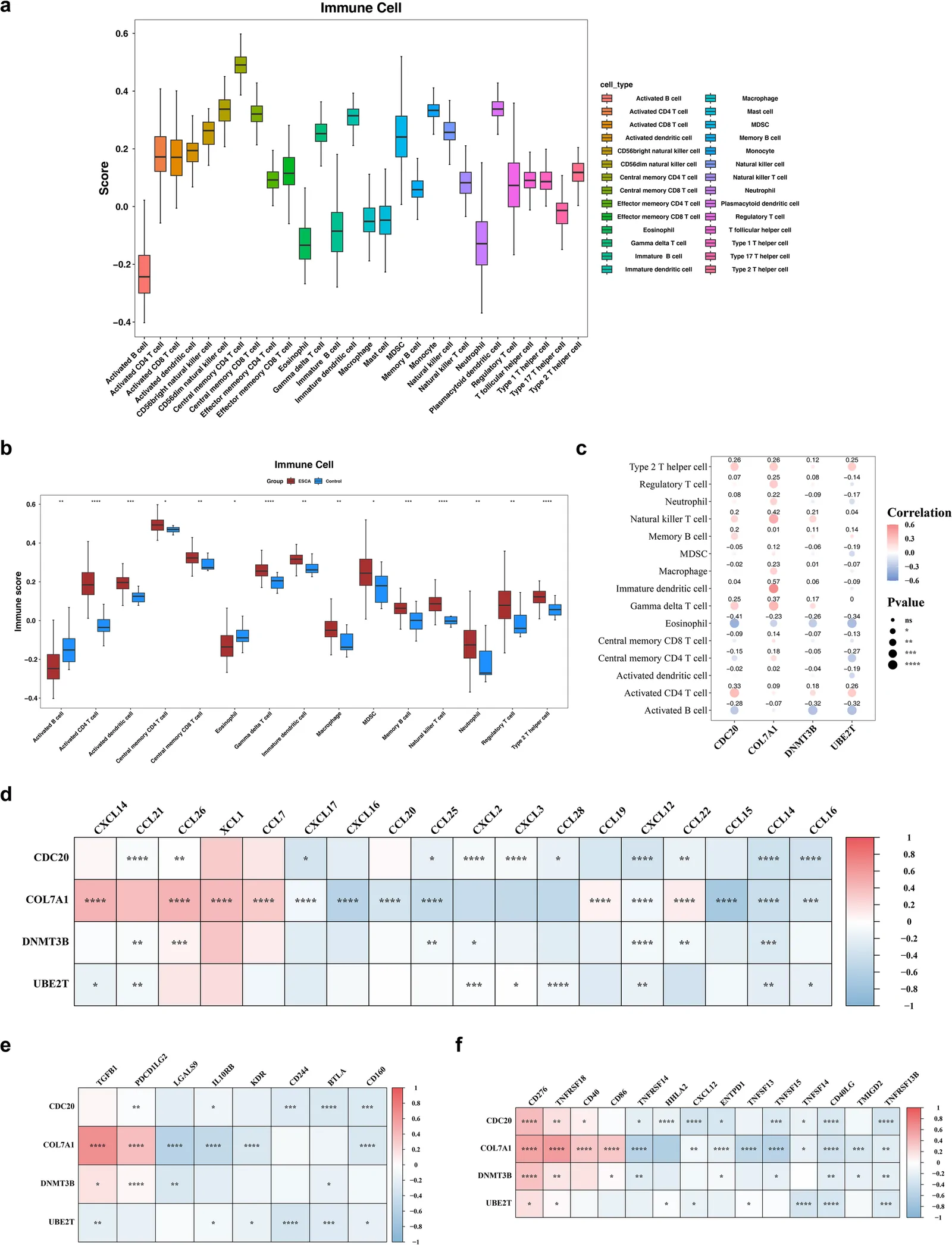
Immune infiltrating cell analysis. **a** Box line plot of immune infiltrating cell profiles. **b** Difference of immune infiltrating cell abundance among disease controls Horizontal coordinates represent immune cells, vertical coordinates represent immune scores of immune cells in the sample, *p < 0.05, **p < 0.01, ***p < 0.001, ****p < 0.0001. **c** biomarker and immune infiltrating cell correlation analysis. Horizontal coordinates indicate genes, vertical coordinates indicate immune cells, and scores indicate correlation; circle size indicates significance; (**d**) Heatmap of biomarker and chemokine correlation Horizontal coordinates indicate chemokine genes, and vertical coordinates indicate biomarkers; (**e**) Heatmap of biomarker and Immunoinhibitor correlation Note: Horizontal coordinates indicate the Immunoinhibitor gene, vertical coordinate indicates biomarker; (**f**) Heat map of biomarker correlation with Immunostimulator Horizontal coordinate indicates Immunostimulator gene, vertical coordinate indicates biomarker *p < 0.05, **p < 0.01, ***p < 0.001, ****p < 0.0001

The box line diagram displayed the distribution proportion of 28 types of immune infiltrating cells in the samples in TCGA-ESCA dataset, with the vertical axis showed the immune infiltration score. We found that central memory CD8 T cells had the highest infiltration score (Fig. 5a). The ESCA and control groups were shown to vary substantially in 15 different kinds of immune cells, such as active CD4 T cells and activated B cells. 13 immune cells out of them had illness scores that were noticeably greater than those of the control group (Fig. 5b). In addition, the findings revealed a significant negative link with eosinophils (*r* = -0.41, *p* < 0.0001) and a robust positive relationship with activated CD4 T cells (*r* = 0.33, *p* < 0.0001) for CDC20. There were positive correlations between COL7A1 and gamma delta T cells, natural killer T cells, and immature dendritic cells (*r* > 0.3, *p* < 0.0001). Activated B cells, UBE2T, and DNMT3B were strongly negatively correlated (*r* < -0.3, *p* < 0.0001). Furthermore, Eosinophil and UBE2T showed a significant negative correlation (*r* = -0.34, *p* < 0.0001) (Fig. 5c, Additional file 1). Notably, CDC20, COL7A1, DNMT3B and UBE2T showed significant correlation with 18 chemokines (CXCL14, CCL21, CCL26, XCL1, CCL7, and so on), 8 immunoinhibitors (such as TGFB1 and PDCD1LG2), 14 immunostimulators (such as CD276, TNFRSF18, CD40, and CD86) (Fig. 5d-f) (|r| > 0.3, *p* < 0.05). For instance, the majority of cell regulatory variables were substantially correlated with COL7A1 (*p* < 0.05). All biomarkers showed a significant correlation (*p* < 0.05) with immunostimulatory factor CD276. Immune cell correlation coefficients only described co-expression trends and could not validate regulatory causality.

### Regulatory network and drugs associated with biomarkers

**Fig. 6:**
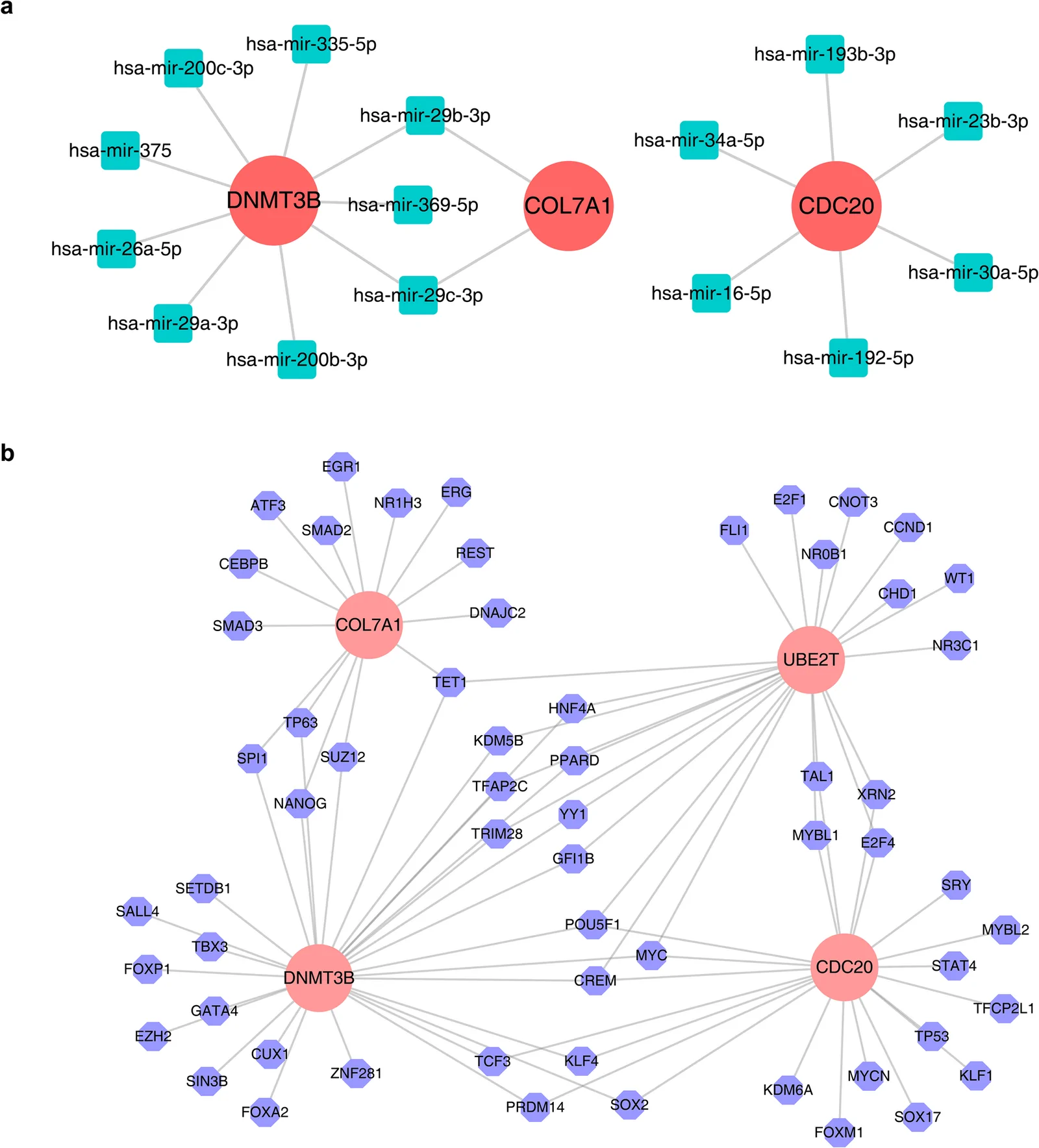
Construction of upstream regulatory networks. **a** miRNA-mRNA network construction, mRNA in red; miRNA in green ; (**b**) Construction of biomarker TF network, mRNA in red, TF in purple

When 28 miRNAs from the miRTarBase database and 120 miRNAs from the TarBase database intersected, a total of 17 miRNAs were found. Based on these findings, a miRNA-mRNA network was constructed, including interactions such as ‘hsa-mir-29b-3p’-COL7A1, ‘hsa-mir-369-5p’-DNMT3B, and ‘hsa-mir-34a-5p’-CDC20 (Fig. 6a). Additionally, TFs associated with CDC20, COL7A1, DNMT3B, and UBE2T were identified, totaling 21, 14, 29, and 23 TFs, respectively. Notable, these TFs included KDM5B, TFAP2C, TRIM28, GFI1B, and POU5F1, etc. The TF-mRNA regulatory network was established by integrating these predicted TFs with biomarkers, revealing interactions such as TET1-COL7A1, POU5F1-DNMT3B, CHD1-UBE2T, and SRY-CDC20 (Fig. 6b). Overall, these findings highlighted the complex regulatory relationships between miRNAs, TFs, and their target mRNAs, providing deeper insights into the molecular mechanisms underlying gene expression regulation.

**Fig. 7:**
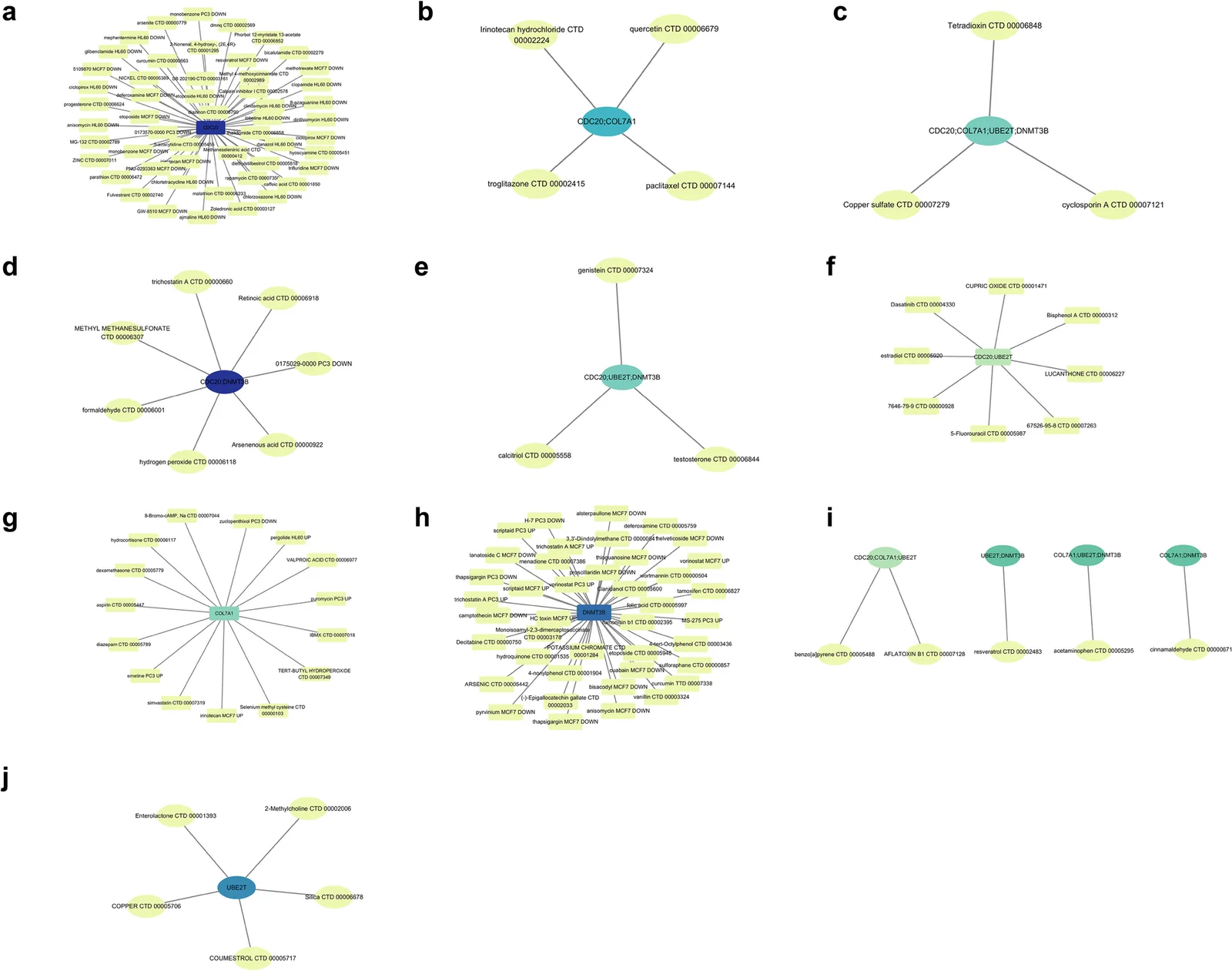
Potential drug prediction networks for biomarkers. **a** Potential therapeutic drugs associated with CDC20; (**b**) Drugs that act in concert with CDC20, COL7A1; (**c**) Drugs that act in concert with CDC20, COL7A1, UBE2T, DNMT3B; (**d**) Drugs that act in concert with CDC20, DNMT3B; (**e**) Drugs that act in concert with CDC20, UBE2T, DNMT3B; (**f**) CDC20, UBE2T co-acting drug; (**g**) Potential therapeutic drugs related to COL7A1;(**h**)Potential therapeutic drugs associated with DNMT3B; (**i**) Potential therapeutic agents related to UBE2T; (**j**)From left to right this is CDC20, COL7A1, UBE2T co-acting drug, UBE2T, DNMT3B co-acting drug, COL7A1, UBE2T, DNMT3B co-acting acting drugs, COL7A1, DNMT3B co-acting drugs

Using DSigDB, potential targeted drugs related to biomarkers were predicted, resulting in the identification of 79 drugs for CDC20, 26 drugs for COL7A1, 67 drugs for DNMT3B, and 23 drugs for UBE2T. Based on the information, these drugs for were used to developed the drug-biomarker networks (Fig. 7). These findings suggested potential therapeutic options for targeting these biomarkers, which could provide valuable insights for further experimental validation and clinical applications. Importantly, miRNA, TF and drug interaction networks were purely bioinformatic predictions without physical binding validation experiments.

### CDC20, COL7A1, DNMT3B and UBE2T expression in ESCA cells

**Fig. 8:**
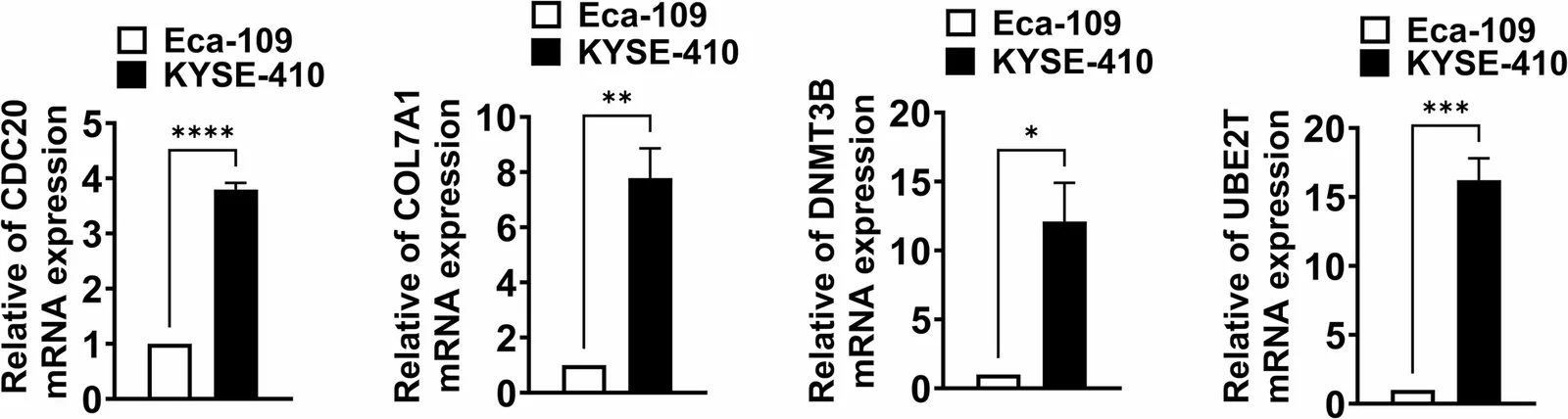
RT-qPCR validation. RT-qPCR validation of CDC20, COL7A1, DNMT3B and UBE2T expression in esophageal cancer cell lines (Eca-109, KYSE-410), *p < 0.05, **p < 0.01, ***p < 0.001, ****p < 0.0001

Based on the qRT-PCR results, ESCA cell lines (Eca-109, KYSE-410) exhibited high expression levels of CDC20, COL7A1, DNMT3B, and UBE2T (*p* < 0.05). The mRNA expression of CDC20, COL7A1, DNMT3B and UBE2T was higher in KYSE-410 cells than in the Eca-109 cell line (Fig. 8). The findings demonstrate that both Eca-109 and KYSE-410 cells had noticeably higher mRNA levels of the four markers mentioned above, indicating that the biomarkers could be valuable prognostic factors and play a significant part in ESCA growth. Only mRNA expression differences were verified; functional regulatory roles of the four genes were not validated via knockout or overexpression assays.

## Discussion

In this study, we systematically identified four RBP-associated biomarkers—CDC20, COL7A1, DNMT3B, and UBE2T—in ESCA through integrated differential expression analysis, PPI network construction, machine learning, and ROC validation. Notably, despite continued advances in medical technology, ESCA remains associated with high morbidity and mortality, and effective therapeutic strategies are still urgently needed [42, 43]. In this context, our findings address this gap by providing candidate targets for mechanistic investigation and potential clinical translation [44]. K-M survival analyses revealed statistical correlations between elevated DNMT3B, UBE2T and poor OS/DSS outcomes, and this expression trend was consistent with RT-qPCR data obtained from ESCA cell lines, which supported the clinical relevance of the four screened genes. Importantly, both Eca-109 and KYSE-410 cells exhibited elevated mRNA levels of the four biomarkers; KYSE-410 (poorly differentiated, highly invasive) showed higher expression abundance, which suggested a correlative link between the expression levels of these genes and tumor malignant phenotypes.

Cell division cycle protein 20 (CDC20), an oncogenic regulator, activates the anaphase-promoting complex/cyclosome (APC/C), thereby promoting sister chromatid separation—a critical step in mitosis [45]. CDC20 is highly expressed across multiple tumor types, including pancreatic cancer [46], breast cancer [47], hepatocellular carcinoma [48], and gastric cancer [49]. and this overexpression exhibited consistent correlations with tumor cell invasion, migration, and proliferation. In ESCC specifically, elevated CDC20 expression has been linked to unfavorable prognosis, tumor metastasis, and disease recurrence [50]. Consistent with these reports, our study identified CDC20 as a significantly upregulated biomarker in ESCA, further supporting its potential relevance to ESCA malignant progression and highlighting its value as a candidate therapeutic target. As a transcript susceptible to post-transcriptional modulation by RBPs, CDC20 mRNA stability and translation could be altered via RBP-dependent transcript regulation, which implied that elevated CDC20 in ESCA might correlate with abnormal RBP activity. Nevertheless, current evidence only demonstrates transcriptional-level correlations between RBPs and CDC20 expression across various malignancies. Direct biochemical and cellular experiments are still lacking to validate their causal functional interactions, such as physical binding between RBPs and CDC20 mRNA, and RBP-mediated regulation of CDC20 mRNA stability and translation. Although previous studies observed post-transcriptional modification of CDC20 transcripts, transcriptome correlation data alone failed to verify that abnormal RBP expression was the critical factor associated with CDC20 overexpression and ESCA progression.

Collagen type VII alpha 1 chain (COL7A1) encodes type VII collagen—a protein with 118 exons that plays a crucial role in fibril anchoring at the dermo-epidermal junction. Mutations affecting the triple-helical domain, hinge region, or promoter region can disrupt its structural function [51]. Emerging evidence implicates COL7A1 in cancer prognosis: survival analysis has demonstrated its influence on outcomes in laryngeal squamous cell carcinoma (LSCC) [52], while somatic COL7A1 mutations are associated with worse OS in colorectal cancer patients [53]. Loss of functional COL7A1 also enhances the invasive and metastatic behavior of cutaneous squamous cell carcinomas [54], and knockdown of COL7A1 significantly impairs the migratory capacity of clear cell renal cell carcinoma cells, underscoring its contribution to tumor aggressiveness [55]. In ESCA, high COL7A1 expression correlates with lymphovascular invasion, depth of tumor invasion, and poor prognosis [56]. The overexpression of COL7A1 observed in gastric cancer has further been proposed to facilitate distant metastasis through remodeling of the tumor microenvironment [57]. Collectively, clinical data supported COL7A1 as a potential ESCC prognostic marker for risk stratification and therapy research. Our transcriptomic data grouped COL7A1 into RBP-correlated gene sets, suggesting its expression may be modulated post-transcriptionally by RBPs. However, only transcriptional co-expression trends were observed, without biochemical proof of RBP-COL7A1 mRNA binding. We therefore could not conclude that aberrant RBP profiles correlated with COL7A1 overexpression and ECM remodeling in ESCA.

DNA (cytosine-5)-methyltransferase 3B (DNMT3B) is a de novo DNA methyltransferase that drives aberrant DNA methylation, thereby dysregulating the expression of both tumor suppressor genes and oncogenes [58]. Elevated DNMT3B expression has been documented in bladder cancer, where its inhibition suppresses tumor cell migration and invasion [59], as well as in endometrial cancer, where high protein levels correlate with poor prognosis [60]. Across multiple additional malignancies—including hepatocellular carcinoma [61] and colorectal carcinoma [62]. —DNMT3B overexpression is closely linked to oncogene silencing through hypermethylation, further supporting its role as a driver of tumor progression. These findings align with our observation that DNMT3B expression was significantly elevated in the ESCA group relative to adjacent normal tissues. In ESCA specifically, DNMT3B knockdown suppresses tumor cell proliferation, while its overexpression markedly promotes growth [63], a pattern consistent with our results and reinforcing its candidacy as both a diagnostic and prognostic biomarker in this malignancy. Network analysis detected correlations between DNMT3B and multiple RBPs, hinting RBPs may affect DNMT3B transcripts post-transcriptionally. Still, we only possessed transcriptome correlation results, lacking RIP/RNA pull-down evidence to validate RBP-mRNA physical interactions. We could not confirm that abnormal RBPs correlated with DNMT3B-dependent epigenetic changes; this relationship required functional validation.

The ubiquitin-conjugating enzyme E2T (UBE2T), a member of the E2 ubiquitin-conjugating enzyme family, is essential for FANCD2 monoubiquitination and plays a key role in DNA damage repair [64]. UBE2T is broadly overexpressed across cancer types and promotes metastasis, tumorigenesis, and proliferation in an E2 activity-dependent manner. In hepatocellular carcinoma, UBE2T accelerates tumor growth by facilitating ubiquitination and proteasomal degradation of p53; [65] in osteosarcoma, it drives cell invasion and proliferation via the PI3K/AKT pathway; [66] and in bladder and prostate cancers, it promotes epithelial-mesenchymal transition (EMT) and cancer cell proliferation [67, 68]. In the context of ESCA, a prior study in 90 ESCC patients reported an association between UBE2T expression and disease-free survival (DFS), though no significant correlation with OS was observed [69]. Our K-M curves showed low UBE2T correlated with better OS/DSS across broader ESCA cohorts, possibly due to differences in patient groups and follow-up duration. Collectively, combined data identified UBE2T as a candidate prognostic marker needing larger cohort verification. In addition, UBE2T appeared in our RBP correlation network, which suggested its mRNA stability and translation might be modulated by RBPs, potentially forming an indirect link between RBP dysregulation and genomic instability in ESCA.

In the present study, ROC analysis confirmed that CDC20, COL7A1, DNMT3B, and UBE2T achieved AUC values exceeding 0.7 in both datasets, consistent with prior literature reporting their elevated expression in ESCA. Mechanistically, CDC20 is closely associated with key oncogenic pathways—including the cell cycle, p53 signaling, and spliceosome—in ESCA, and may promote disease progression by modulating cell cycle progression and tumor stemness, positioning it as both a predictive biomarker and a candidate therapeutic target [70, 71]. High COL7A1 expression correlates significantly with tumor invasion depth, lymphovascular invasion, and poorer five-year survival in ESCA, underscoring its role in malignant progression [56]. Similarly, DNMT3B promotes cancer cell proliferation and migration through its targeting interaction with miR-129-2-3p and modulation of cell cycle-related proteins, and is directly linked to unfavorable prognosis in ESCA patients; notably, rhein has been shown to suppress tumor proliferation by downregulating DNMT3B expression, suggesting its therapeutic potential [63, 72]. In ESCA, UBE2T expression is closely associated with the p53 signaling pathway, cell cycle regulation, DNA replication, and the Fanconi anemia pathway, and correlates with poor prognosis; a UBE2T-based gene signature model has demonstrated efficacy in predicting patient outcomes, with individuals in the low-risk group showing enhanced sensitivity to chemotherapy [69]. Collectively, the four transcripts represented candidate prognostic markers and therapeutic targets for ESCA.

To further elucidate the molecular mechanisms underlying biomarker function in ESCA carcinogenesis, GSEA enrichment analyses were performed for all four biomarkers. COL7A1 was found to be associated with the nitrogen metabolism pathway. Nitrogen is an essential element for cellular growth and division, with amino acids and nucleotides serving as its primary intracellular sources [73]. To sustain rapid proliferation and high metabolic demands, tumor cells frequently reprogram nitrogen metabolism by upregulating nitrogen uptake, enhancing the activity of nitrogen-metabolizing enzymes, and optimizing nitrogen flux—thereby creating a growth-supportive microenvironment [74, 75]. Given that ESCA is closely associated with chronic inflammation and immune dysregulation, the enrichment of COL7A1 in the nitrogen metabolism pathway suggests that it may potentially contribute to dysregulation of the blood urea nitrogen/creatinine ratio (BUN/Cr), a nitrogen-containing plasma component whose elevation has been linked to exacerbated inflammation in ESCA patients [76, 77] —though direct mechanistic evidence for this association remains to be established. Targeting nitrogen metabolic pathways therefore represents a potentially promising anti-cancer therapeutic strategy worthy of further investigation.

Meanwhile, other core pathway enrichments were also observed in the remaining biomarkers. CDC20 and UBE2T were co-enriched in the DNA mismatch repair (MMR) pathway, while DNMT3B showed high enrichment in the endocytosis pathway. MMR is a conserved biological mechanism responsible for correcting base mismatches, insertions, and deletions arising during DNA replication, thereby preserving genomic stability [78]. MMR deficiency leads to microsatellite instability (MSI), a hallmark feature of endometrial and gastric cancers associated with elevated tumorigenesis risk [79, 80]. MMR-deficient tumors frequently accumulate high neoantigen burdens; while these neoantigens may partially activate immune responses, tumor cells can nonetheless achieve immune evasion through overexpression of immune checkpoint molecules such as PD-L1. Given that CDC20 and UBE2T regulate DNA replication and RNA splicing, their co-enrichment in the MMR pathway suggests potential roles in genomic instability and ESCA progression. Regarding endocytosis—the cellular process by which extracellular materials are internalized via plasma membrane-derived vesicles—dysregulation of this pathway has been implicated in carcinogenesis through multiple mechanisms: promoting NSCLC cell migration and invasion via the endocytosis-recycling circuit [81], supporting KRAS-mutant tumor cell survival through macropinocytosis [82], and facilitating immune evasion by modulating tumor-associated antigen presentation [83, 84]. The high enrichment score of DNMT3B in the endocytosis pathway suggests that it may enhance therapeutic efficacy in ESCA through receptor-mediated endocytosis [85], offering novel avenues for targeted drug delivery and metabolic disruption of tumor cells.

However, in the face of the high mortality rate associated with ESCA, developing novel therapeutic strategies remains a significant challenge, largely due to the complexity of the tumor microenvironment. To address this, we assessed the relationship between the four biomarkers and tumor-infiltrating immune cells. Eosinophils are key components of the innate immune system, classically involved in antiparasitic responses and allergic reactions [86]. In tumors, eosinophils exhibit dual functionality: they can promote tumor growth by secreting soluble mediators that drive angiogenesis and stromal remodeling and by creating an immunosuppressive microenvironment [87, 88], as demonstrated in cervical cancer [89], ovarian cancer [90], and Hodgkin lymphoma [91]. Conversely, eosinophils also exert antitumor effects—directly lysing tumor cells via cytotoxic mediators in colorectal cancer [92], inhibiting tumor progression through ROS release and microenvironmental modulation in an ESCC mouse model [93], enhancing NK cell cytotoxicity against liver metastases [94], and inversely correlating with metastasis in small cell lung cancer [95]. In the present study, eosinophil infiltration in ESCA was significantly negatively correlated with CDC20 and UBE2T expression, and patients with low UBE2T expression demonstrated markedly better survival outcomes. This transcriptional association implies that elevated CDC20 and UBE2T may suppress eosinophil-mediated anti-tumor immunity within the ESCC microenvironment. However, clinical and preclinical studies have reported context-dependent dual biological roles of eosinophils in ESCC. Multiple retrospective patient cohorts and murine functional experiments validate the anti-tumor capacity of eosinophils: abundant intratumoral eosinophil infiltration is linked to less lymphatic metastasis and favorable survival for ESCC patients receiving concurrent chemoradiotherapy, while activated eosinophils induce cancer cell apoptosis through degranulation and reactive oxygen species secretion [93, 96]. By contrast, systematic reviews focused on esophageal innate immunity demonstrate that eosinophils can switch to pro-tumor phenotypes in advanced lesions remodeled by aberrant cytokine signaling, demonstrating functional plasticity governed by tumor progression stage and microenvironmental stimuli [97]. Since CDC20 and UBE2T were part of RBP-correlated modules, we put forward a tentative hypothesis that altered RBP expression might associate with upregulated CDC20/UBE2T and eosinophil-related immune escape.

Natural killer T (NKT) cells are a specialized immune subpopulation that integrates features of both NK cells and conventional T cells, playing critical roles in tumor immune surveillance and immune evasion [98, 99]. In antitumor immunity, NKT cells rapidly release IL-4 and IFN-γ upon recognition of glycolipid antigens presented by CD1d molecules, enabling direct tumor cell killing. NKT cell infiltration is associated with favorable prognoses in colorectal cancer [100], pancreatic cancer [101], and neuroblastoma [102], and NKT cells can indirectly reshape the tumor microenvironment by modulating MDSCs and dendritic cells [103]. However, in immunosuppressive microenvironments, NKT cells may acquire pro-tumorigenic functions—secreting IL-13, promoting MDSC recruitment, and suppressing antitumor immunity [104]. In this study, COL7A1 expression was significantly positively correlated with NKT cell infiltration in ESCA specimens, suggesting that COL7A1 may influence the antitumor activity of NKT cells and thereby modulate disease progression—a relationship that warrants functional validation in future studies. Given that COL7A1 was identified as part of the RBP-associated gene network, RBP-mediated regulation of COL7A1 expression may indirectly shape NKT cell behavior within the ESCA tumor microenvironment, warranting further mechanistic investigation.

Furthermore, activated B lymphocytes play a multifaceted role in the tumor microenvironment, simultaneously promoting antitumor immunity and, in certain contexts, facilitating immune evasion. B cells contribute to anticancer immunity by generating tumor-specific antibodies that mark tumor cells for phagocytic clearance and by secreting cytokines that activate CD4 + T cells and NK cells [105]. Their presence in the tumor microenvironment enhances immune surveillance through TIL formation and is associated with improved survival and reduced recurrence in ovarian cancer, NSCLC, and cervical cancer [106–108]. Paradoxically, activated B cells can also promote tumor progression by secreting immunosuppressive cytokines such as IL-10, fostering Treg expansion, or acquiring immunosuppressive properties upon tumor microenvironment recruitment [105]. In this study, DNMT3B and UBE2T expression were significantly negatively correlated with activated B cell infiltration, suggesting that the suppressive role of activated B cells in ESCA may be attenuated by high expression of these two biomarkers—a finding that highlights the importance of dissecting B cell functional states in ESCA and may inform future B cell-targeted immunotherapy strategies. Since DNMT3B and UBE2T are embedded within the RBP-associated regulatory network identified in this study, RBP-driven dysregulation of these genes may represent one mechanism by which the tumor microenvironment suppresses B cell antitumor activity in ESCA.

## Conclusions

In summary, this study identified four RBP-associated biomarkers—CDC20, COL7A1, DNMT3B, and UBE2T—in ESCA through integrated bioinformatics analysis. ROC analysis confirmed strong diagnostic value for all four markers (AUC > 0.7), and K-M survival curves demonstrated that elevated DNMT3B and UBE2T expression was significantly associated with worse OS and DSS (*P* < 0.05). Based on these biomarkers, regulatory network construction, immune infiltration profiling, and functional enrichment analyses provided new mechanistic insights into ESCA diagnosis and prognosis. Nevertheless, several limitations should be acknowledged. First, the present study solely relies on public transcriptomic datasets for correlation analysis. All findings generated are merely bioinformatic correlation predictions, which cannot verify direct causal molecular regulatory relationships. Accordingly, relevant conclusions should be interpreted with caution and require further validation through cellular and animal functional experiments in subsequent research. Second, while RT-qPCR confirmed elevated mRNA expression of the four genes in Eca-109 and KYSE-410 cell lines, functional experiments—including knockdown or overexpression studies—were not performed, precluding causal inference regarding their roles in ESCA pathogenesis. Third, the selection and performance of bioinformatics algorithms may introduce methodological variability that affects result interpretation. Fourth, transcriptome correlation analyses from our cohort suggest that upregulated CDC20 and UBE2T may attenuate eosinophil-mediated anti-tumor effects in esophageal cancer. However, previous clinical cohort studies have drawn inconsistent conclusions regarding the biological functions of eosinophils in ESCC, as eosinophils exert context-dependent dual roles within the tumor microenvironment. For this reason, this hypothesis requires cautious interpretation, and functional experiments are still needed to validate the regulatory relationship between CDC20/UBE2T and eosinophils. To overcome these limitations, future studies should integrate in vitro functional assays, in vivo animal models, and prospective clinical cohorts to elucidate the specific mechanisms by which these four biomarkers contribute to ESCA development and progression, ultimately advancing their translational potential.

## Supplementary Information

Additional file 1. Correlation analysis results between biomarkers and immune infiltrating cells.

## Data Availability

No datasets were generated or analysed during the current study.

## Abbreviations

APC/C: Anaphase-promoting complex/cyclosome
AUC: Area under the curve
BMI: Body mass index
BP: Biological process
BUN/Cr: Blood urea nitrogen/creatinine ratio
CC: Cellular component
ccRCC: Clear cell renal cell carcinoma
CDC20: Cell division cycle protein 20
cDNA: Complementary DNA
COL7A1: Collagen type VII alpha 1 chain
DEGs: Differentially expressed genes
DFS: Disease-free survival
DMEM: Dulbecco’s modified Eagle medium
DNMT3B: DNA (cytosine-5)-methyltransferase 3B
DSS: Disease-specific survival
EAC: Esophageal adenocarcinoma
EC: Esophageal cancer
ECM: Extracellular matrix
EMT: Epithelial-mesenchymal transition
ESCA: Esophageal cancer
ESCC: Esophageal squamous cell carcinoma
FBS: Foetal bovine serum
FC: Fold change
GEO: Gene Expression Omnibus
GO: Gene Ontology
GSVA: Gene set variation analysis
GSEA: Gene set enrichment analysis
IFN-γ: Interferon-gamma
IL: Interleukin
K-M: Kaplan-Meier
KEGG: Kyoto Encyclopedia of Genes and Genomes
LASSO: Least absolute shrinkage and selection operator
LSCC: Laryngeal squamous cell carcinoma
LUSC: Lung squamous cell carcinoma
MDSCs: Myeloid-derived suppressor cells
MF: Molecular function
miRNA: MicroRNA
MMR: Mismatch repair
MSI: Microsatellite instability
mRNA: Messenger RNA
MSigDB: Molecular Signatures Database
NK: Natural killer
NKT: Natural killer T
NSCLC: Non-small cell lung cancer
OS: Overall survival
PPI: Protein-protein interaction
RBP: RNA-binding protein
RBPRGs: RNA-binding protein-related genes
RBPTD: RNA-binding proteins target database
ROC: Receiver operating characteristic
ROS: Reactive oxygen species
RT-qPCR: Quantitative reverse transcription-polymerase chain reaction
ssGSEA: Single-sample gene set enrichment analysis
SVM-RFE: Support vector machine-recursive feature elimination
TCGA: The Cancer Genome Atlas
TF: Transcription factor
TIL: Tumor-infiltrating lymphocyte
TISIDB: Tumor-Immune System Interaction Database
UCSC: University of California Santa Cruz
UBE2T: Ubiquitin-conjugating enzyme E2T
UTR: Untranslated region
WHO: World Health Organisation

## References

[1] Huang YJ, Rieder J, Tan KV, Tenditnaya A, Vojnovic B, Gorpas D, et al. Targeting c-MET for Endoscopic Detection of Dysplastic Lesions within Barrett’s Esophagus Using EMI-137 Fluorescence Imaging. Clin Cancer Res. 2025;31:98–109. 10.1158/1078-0432.Ccr-24-1522. PMC11701434 39513952

[2] Sung H, Ferlay J, Siegel RL, Laversanne M, Soerjomataram I, Jemal A, et al. Global Cancer Statistics 2020: GLOBOCAN Estimates of Incidence and Mortality Worldwide for 36 Cancers in 185 Countries. CA Cancer J Clin. 2021;71:209–49. 10.3322/caac.21660. 33538338

[3] Yang D, Peng D, Zhou Y, Qiang Z, Wan L, Fan X, et al. Alpha-Momorcharin, a type I ribosome inactivating protein, induced apoptosis of hepatocellular carcinoma SK-HEP-1 cells through mitochondrial pathway. Nat Prod Res. 2023;1–11. 10.1080/14786419.2023.2295915. 38126176

[4] Chen H, Shi X, Ren L, Zhuo H, Zeng L, Qin Q, et al. Identification of the miRNA-mRNA regulatory network associated with radiosensitivity in esophageal cancer based on integrative analysis of the TCGA and GEO data. BMC Med Genomics. 2022;15:249. 10.1186/s12920-022-01392-9. PMC9714096 36456979

[5] Boerner T, Carr RA, Hsu M, Michel A, Tan KS, Vos E, et al. Incidence and management of esophageal cancer recurrence to regional lymph nodes after curative esophagectomy. Int J Cancer. 2023;152:2109–22. 10.1002/ijc.34417. PMC10006335 36573352

[6] Ma Z, Zhu T, Wang H, Wang B, Fu L, Yu G. E2F1 Reduces Sorafenib’s Sensitivity of Esophageal Carcinoma Cells via Modulating the miR-29c-3p/COL11A1 Signaling Axis. Curr Mol Pharmacol. 2024;17:e060323214360. 10.2174/1874467216666230306101653. 36876834

[7] Gou H, Yang H, Cheng J, He S, Luo C, Chen X, et al. YY2 Serves as a Novel Prognostic Biomarker Correlated with Immune Microenvironment and Glycolysis in Esophageal Carcinoma. Curr Genomics. 2025;26:312–28. 10.2174/0113892029358348250124064940. PMC12606659 41235100

[8] Ba Y, Gu X. Using single-cell RNA sequencing and bulk RNA sequencing data to reveal a correlation between smoking and neutrophil activation in esophageal carcinoma patients. Environ Toxicol. 2024;39:4689–99. 10.1002/tox.24312. 38700434

[9] Xue C, Cai X, Jia J. Long Non-coding RNA Double Homeobox A Pseudogene 8: A Novel Oncogenic Propellant in Human Cancer. Front Cell Dev Biol. 2021;9:709069. 10.3389/fcell.2021.709069. PMC8495153 34631702

[10] Song Y, An W, Wang H, Gao Y, Han J, Hao C, et al. LRH1 Acts as an Oncogenic Driver in Human Osteosarcoma and Pan-Cancer. Front Cell Dev Biol. 2021;9:643522. 10.3389/fcell.2021.643522. PMC8005613 33791301

[11] Hu L, Cao H, Zheng L, Li R. BBOX1-AS1 Activates Hedgehog Signaling Pathway to Facilitate the Proliferation and Stemness of Esophageal Squamous Cell Carcinoma Cells via miR-506-5p/EIF5A/PTCH1 Axis. Curr Mol Pharmacol. 2023;16:894–904. 10.2174/1874467216666230130132927. 36717994

[12] Li P, Gao X, Huang D, Gu X. Identification and Characterization of Prognostic Macrophage Subpopulations for Human Esophageal Carcinoma. Curr Med Chem. 2025;32:123–35. 10.2174/0109298673284207240108105724. 38362682

[13] Velázquez-Cruz A, Baños-Jaime B, Díaz-Quintana A, De la Rosa MA, Díaz-Moreno I. Post-translational Control of RNA-Binding Proteins and Disease-Related Dysregulation. Front Mol Biosci. 2021;8:658852. 10.3389/fmolb.2021.658852. PMC8111222 33987205

[14] Gebauer F, Schwarzl T, Valcárcel J, Hentze MW. RNA-binding proteins in human genetic disease. Nat Rev Genet. 2021;22:185–98. 10.1038/s41576-020-00302-y. 33235359

[15] Mir C, Garcia-Mayea Y, ME LL. Targeting the undruggable: RNA-binding proteins in the spotlight in cancer therapy. Semin Cancer Biol. 2022;86:69–83. 10.1016/j.semcancer.2022.06.008. 35772609

[16] Zhao S, Liu Q, Li J, Hu C, Cao F, Ma W, et al. Construction and Validation of Prognostic Regulation Network Based on RNA-Binding Protein Genes in Lung Squamous Cell Carcinoma. DNA Cell Biol. 2021;40:1563–83. 10.1089/dna.2021.0145. 34931870

[17] Lou N, Meng X, Yu T, Li W, Lv X, Han W, et al. Prognostic Value and Immunological Role of POP7 in Clear Cell Renal Cell Carcinoma. Pharmgenomics Pers Med. 2024;17:521–34. 10.2147/pgpm.S469247. PMC11608043 39620194

[18] Zhang Z, Yu C, Li Y, Jiang L, Zhou F. Utility of SAM68 in the progression and prognosis for bladder cancer. BMC Cancer. 2015;15:364. 10.1186/s12885-015-1367-x. PMC4425873 25944080

[19] Li Z, Yu CP, Zhong Y, Liu TJ, Huang QD, Zhao XH, et al. Sam68 expression and cytoplasmic localization is correlated with lymph node metastasis as well as prognosis in patients with early-stage cervical cancer. Ann Oncol. 2012;23:638–46. 10.1093/annonc/mdr290. 21700735

[20] He L, Zhou X, Qu C, Hu L, Tang Y, Zhang Q, et al. Musashi2 predicts poor prognosis and invasion in hepatocellular carcinoma by driving epithelial-mesenchymal transition. J Cell Mol Med. 2014;18:49–58. 10.1111/jcmm.12158. PMC3916117 24305552

[21] Gong P, Wang Y, Gao Y, Gao M, Liu L, Qu P, et al. Msi1 promotes tumor progression by epithelial-to-mesenchymal transition in cervical cancer. Hum Pathol. 2017;65:53–61. 10.1016/j.humpath.2016.12.026. 28088346

[22] Wang J, Zhu L, Zhang Q, Xia T, Yao W, Wei L. LincRNA-P21 knockdown facilitates esophageal squamous cell carcinoma cell progression by upregulating cadherin 5 via YTH domain containing 1. Acta Biochim Biophys Sin (Shanghai). 2023;55:1797–805. 10.3724/abbs.2023154. PMC10686791 37766459

[23] Zhou Y, Xue X, Luo J, Li P, Xiao Z, Zhang W, et al. Circular RNA circ-FIRRE interacts with HNRNPC to promote esophageal squamous cell carcinoma progression by stabilizing GLI2 mRNA. Cancer Sci. 2023;114:3608–22. 10.1111/cas.15899. PMC10475760 37417427

[24] Zhang Y, Chen C, Liu Z, Guo H, Lu W, Hu W, et al. PABPC1-induced stabilization of IFI27 mRNA promotes angiogenesis and malignant progression in esophageal squamous cell carcinoma through exosomal miRNA-21-5p. J Exp Clin Cancer Res. 2022;41:111. 10.1186/s13046-022-02339-9. PMC8962095 35346324

[25] Wang H. Network pharmacology- and molecular docking-based approaches to unveil the pharmacological mechanisms of dihydroartemisinin against esophageal carcinoma. Front Genet. 2022;13:1017520. 10.3389/fgene.2022.1017520. PMC9732420 36506308

[26] Yang P, Zhang P, Zhang S, RNA-Binding Protein. MEX3A Interacting with DVL3 Stabilizes Wnt/β-Catenin Signaling in Endometrial Carcinoma. Int J Mol Sci. 2022;24. 10.3390/ijms24010592. PMC9820120 36614043

[27] Love MI, Huber W, Anders S. Moderated estimation of fold change and dispersion for RNA-seq data with DESeq2. Genome Biol. 2014;15:550. 10.1186/s13059-014-0550-8. PMC4302049 25516281

[28] Chen LP, Wang H, Zhang Y, Chen QX, Lin TS, Liu ZQ, et al. Robust analysis of novel mRNA-lncRNA cross talk based on ceRNA hypothesis uncovers carcinogenic mechanism and promotes diagnostic accuracy in esophageal cancer. Cancer Manag Res. 2019;11:347–58. 10.2147/cmar.S183310. PMC6312067 30643460

[29] Liu M, Zhao Y, Xiao Z, Zhou R, Chen X, Cui S, et al. CD39-Expressing CD8(+) T Cells as a New Molecular Marker for Diagnosis and Prognosis of Esophageal Squamous Cell Carcinoma. Cancers (Basel). 2023;15. 10.3390/cancers15041184. PMC9954671 36831526

[30] Gustavsson EK, Zhang D, Reynolds RH, Garcia-Ruiz S, Ryten M. ggtranscript: an R package for the visualization and interpretation of transcript isoforms using ggplot2. Bioinformatics. 2022;38:3844–6. 10.1093/bioinformatics/btac409. PMC9344834 35751589

[31] Gu Z, Hübschmann D. Make Interactive Complex Heatmaps in R. Bioinformatics. 2022;38:1460–2. 10.1093/bioinformatics/btab806. PMC8826183 34864868

[32] Meng XW, Cheng ZL, Lu ZY, Tan YN, Jia XY, Zhang M. MX2: Identification and systematic mechanistic analysis of a novel immune-related biomarker for systemic lupus erythematosus. Front Immunol. 2022;13:978851. 10.3389/fimmu.2022.978851. PMC9433551 36059547

[33] Shannon P, Markiel A, Ozier O, Baliga NS, Wang JT, Ramage D, et al. Cytoscape: a software environment for integrated models of biomolecular interaction networks. Genome Res. 2003;13:2498–504. 10.1101/gr.1239303. PMC403769 14597658

[34] Van Essen DC. Cortical cartography and Caret software. NeuroImage. 2012;62:757–64. 10.1016/j.neuroimage.2011.10.077. PMC3288593 22062192

[35] Robin X, Turck N, Hainard A, Tiberti N, Lisacek F, Sanchez JC, et al. pROC: an open-source package for R and S + to analyze and compare ROC curves. BMC Bioinformatics. 2011;12:77. 10.1186/1471-2105-12-77. PMC3068975 21414208

[36] Zhang H, Meltzer P, Davis S. RCircos: an R package for Circos 2D track plots. BMC Bioinformatics. 2013;14:244. 10.1186/1471-2105-14-244. PMC3765848 23937229

[37] Hu Y, Wu Y, Gan F, Jiang M, Chen D, Xie M, et al. Identification of Potential Therapeutic Target Genes in Osteoarthritis. Evid Based Complement Alternat Med. 2022;2022:8027987. 10.1155/2022/8027987. PMC9392645 35996406

[38] Guo L, Yuan M, Jiang S, Jin G, Li P. Expression of pyroptosis-associated genes and construction of prognostic model for thyroid cancer. Transl Cancer Res. 2023;12:3360–83. 10.21037/tcr-23-810. PMC10774054 38193001

[39] Charoentong P, Finotello F, Angelova M, Mayer C, Efremova M, Rieder D, et al. Pan-cancer Immunogenomic Analyses Reveal Genotype-Immunophenotype Relationships and Predictors of Response to Checkpoint Blockade. Cell Rep. 2017;18:248–62. 10.1016/j.celrep.2016.12.019. 28052254

[40] Feng Y, Tao F, Qiao H, Tang H. A pan-cancer analysis of ABI3BP: a potential biomarker for prognosis and immunoinfiltration. Front Oncol. 2023;13:1159725. 10.3389/fonc.2023.1159725. PMC10183607 37197424

[41] Skoufos G, Kakoulidis P, Tastsoglou S, Zacharopoulou E, Kotsira V, Miliotis M, et al. TarBase-v9.0 extends experimentally supported miRNA-gene interactions to cell-types and virally encoded miRNAs. Nucleic Acids Res. 2024;52:D304–10. 10.1093/nar/gkad1071. PMC10767993 37986224

[42] Morgan E, Soerjomataram I, Rumgay H, Coleman HG, Thrift AP, Vignat J, et al. The Global Landscape of Esophageal Squamous Cell Carcinoma and Esophageal Adenocarcinoma Incidence and Mortality in 2020 and Projections to 2040: New Estimates From GLOBOCAN 2020. Gastroenterology. 2022;163:649–e658642. 10.1053/j.gastro.2022.05.054. 35671803

[43] Yang L, Jin H, Xie XL, Cao YT, Liu ZH, Li N, et al. Endoscopic resections for superficial esophageal squamous cell epithelial neoplasia: focus on histological discrepancies between biopsy and resected specimens. BMC Gastroenterol. 2021;21:114. 10.1186/s12876-021-01694-9. PMC7941920 33750308

[44] Mimura K, Yamada L, Ujiie D, Hayase S, Tada T, Hanayama H, et al. Immunotherapy for esophageal squamous cell carcinoma: a review. Fukushima J Med Sci. 2018;64:46–53. 10.5387/fms.2018-09. PMC6141453 30058598

[45] Hsieh CC, Yang CY, Peng B, Ho SL, Tsao CH, Lin CK, et al. Allyl Isothiocyanate Suppresses the Proliferation in Oral Squamous Cell Carcinoma via Mediating the KDM8/CCNA1 Axis. Biomedicines. 2023;11. 10.3390/biomedicines11102669. PMC10604360 37893043

[46] Zhang Y, Xue YB, Li H, Qiu D, Wang ZW, Tan SS. Inhibition of Cell Survival by Curcumin Is Associated with Downregulation of Cell Division Cycle 20 (Cdc20) in Pancreatic Cancer Cells. Nutrients. 2017;9. 10.3390/nu9020109. PMC5331540 28165402

[47] HS, LD, CZ. REC8 inhibits proliferation, migration and invasion of breast cancer cells by targeting CDC20. Mol Med Rep. 2022;26. 10.3892/mmr.2022.12751. PMC9178687 35616161

[48] Yang G, Wang G, Xiong Y, Sun J, Li W, Tang T, et al. CDC20 promotes the progression of hepatocellular carcinoma by regulating epithelial–mesenchymal transition. Mol Med Rep. 2021;24. 10.3892/mmr.2021.12122. PMC8127032 33907851

[49] Kim JM, Sohn HY, Yoon SY, Oh JH, Yang JO, Kim JH, et al. Identification of gastric cancer-related genes using a cDNA microarray containing novel expressed sequence tags expressed in gastric cancer cells. Clin Cancer Res. 2005;11:473–82. 15701830

[50] Du Y, Zhang M, Liu X, Li Z, Hu M, Tian Y, et al. CDC20 promotes bone formation via APC/C dependent ubiquitination and degradation of p65. EMBO Rep. 2021;22:e52576. 10.15252/embr.202152576. PMC8419691 34382737

[51] Ham KA, Aung-Htut MT, Fletcher S, Wilton SD. Nonsequential Splicing Events Alter Antisense-Mediated Exon Skipping Outcome in COL7A1. Int J Mol Sci. 2020;21. 10.3390/ijms21207705. PMC7590164 33081018

[52] Li H, Bo S, Guo Y, Wang T, Pan Y. Identification of hub genes and key modules in laryngeal squamous cell carcinoma. Transl Cancer Res. 2024;13:3771–82. 10.21037/tcr-24-104. PMC11319952 39145051

[53] Heczko L, Liška V, Vyčítal O, Fiala O, Šůsová S, Hlaváč V, et al. Targeted panel sequencing of pharmacogenes and oncodrivers in colorectal cancer patients reveals genes with prognostic significance. Hum Genomics. 2024;18:83. 10.1186/s40246-024-00644-2. PMC11264515 39030589

[54] Martins VL, Vyas JJ, Chen M, Purdie K, Mein CA, South AP, et al. Increased invasive behaviour in cutaneous squamous cell carcinoma with loss of basement-membrane type VII collagen. J Cell Sci. 2009;122:1788–99. 10.1242/jcs.042895. PMC2684833 19435799

[55] Koca D, Séraudie I, Jardillier R, Cochet C, Filhol O, Guyon L. COL7A1 Expression Improves Prognosis Prediction for Patients with Clear Cell Renal Cell Carcinoma Atop of Stage. Cancers (Basel). 2023;15. 10.3390/cancers15102701. PMC10216355 37345040

[56] Kita Y, Mimori K, Tanaka F, Matsumoto T, Haraguchi N, Ishikawa K, et al. Clinical significance of LAMB3 and COL7A1 mRNA in esophageal squamous cell carcinoma. Eur J Surg Oncol. 2009;35:52–8. 10.1016/j.ejso.2008.01.025. 18331784

[57] Oh SE, Oh MY, An JY, Lee JH, Sohn TS, Bae JM, et al. Prognostic Value of Highly Expressed Type VII Collagen (COL7A1) in Patients With Gastric Cancer. Pathol Oncol Res. 2021;27:1609860. 10.3389/pore.2021.1609860. PMC8426344 34512204

[58] Ji Y, Wang Y, Zou J, Liu G, Xia M, Ren J, et al. Methyltransferase DNMT3B promotes colorectal cancer cell proliferation by inhibiting PLCG2. Acta Biochim Biophys Sin (Shanghai). 2024;56:1848–59. 10.3724/abbs.2024117. PMC11693860 39108206

[59] Gagliardi M, Strazzullo M, Matarazzo MR. DNMT3B Functions: Novel Insights From Human Disease. Front Cell Dev Biol. 2018;6:140. 10.3389/fcell.2018.00140. PMC6204409 30406101

[60] Gui T, Liu M, Yao B, Jiang H, Yang D, Li Q, et al. TCF3 is epigenetically silenced by EZH2 and DNMT3B and functions as a tumor suppressor in endometrial cancer. Cell Death Differ. 2021;28:3316–28. 10.1038/s41418-021-00824-w. PMC8630057 34175897

[61] Wu H, Zhang W, Wu Z, Liu Y, Shi Y, Gong J, et al. miR-29c-3p regulates DNMT3B and LATS1 methylation to inhibit tumor progression in hepatocellular carcinoma. Cell Death Dis. 2019;10:48. 10.1038/s41419-018-1281-7. PMC6362005 30718452

[62] Ibrahim ML, Klement JD, Lu C, Redd PS, Xiao W, Yang D, et al. Myeloid-Derived Suppressor Cells Produce IL-10 to Elicit DNMT3b-Dependent IRF8 Silencing to Promote Colitis-Associated Colon Tumorigenesis. Cell Rep. 2018;25:3036–e30463036. 10.1016/j.celrep.2018.11.050. PMC6319669 30540937

[63] Li C, Yu J, Feng Y, Sun X, Sun M, Ni W, et al. Rhein suppresses esophageal cancer development by regulating cell cycle through DNMT3B gene. Med Oncol. 2024;41:153. 10.1007/s12032-024-02359-9. 38743323

[64] Zhang Y, Zhou X, Huang P. Fanconi anemia and ubiquitination. J Genet Genomics. 2007;34:573–80. 10.1016/s1673-8527(07)60065-4. 17643942

[65] Liu LP, Yang M, Peng QZ, Li MY, Zhang YS, Guo YH, et al. UBE2T promotes hepatocellular carcinoma cell growth via ubiquitination of p53. Biochem Biophys Res Commun. 2017;493:20–7. 10.1016/j.bbrc.2017.09.091. 28935368

[66] Wang Y, Leng H, Chen H, Wang L, Jiang N, Huo X, et al. Knockdown of UBE2T Inhibits Osteosarcoma Cell Proliferation, Migration, and Invasion by Suppressing the PI3K/Akt Signaling Pathway. Oncol Res. 2016;24:361–9. 10.3727/096504016x14685034103310. PMC7838603 27712593

[67] Gong YQ, Peng D, Ning XH, Yang XY, Li XS, Zhou LQ, et al. UBE2T silencing suppresses proliferation and induces cell cycle arrest and apoptosis in bladder cancer cells. Oncol Lett. 2016;12:4485–92. 10.3892/ol.2016.5237. PMC5228076 28101210

[68] Wen M, Kwon Y, Wang Y, Mao JH, Wei G. Elevated expression of UBE2T exhibits oncogenic properties in human prostate cancer. Oncotarget. 2015;6:25226–39. 10.18632/oncotarget.4712. PMC4694827 26308072

[69] Wang X, Liu Y, Leng X, Cao K, Sun W, Zhu J, et al. UBE2T Contributes to the Prognosis of Esophageal Squamous Cell Carcinoma. Pathol Oncol Res. 2021;27:632531. 10.3389/pore.2021.632531. PMC8262217 34257599

[70] Liu HS, Guo Q, Yang H, Zeng M, Xu LQ, Zhang QX, et al. SPDL1 Overexpression Is Associated With the 18F-FDG PET/CT Metabolic Parameters, Prognosis, and Progression of Esophageal Cancer. Front Genet. 2022;13:798020. 10.3389/fgene.2022.798020. PMC9157543 35664322

[71] Xu W, Xu J, Wang Z, Jiang Y. Weighted Gene Correlation Network Analysis Identifies Specific Functional Modules and Genes in Esophageal Cancer. J Oncol. 2021;2021:8223263. 10.1155/2021/8223263. PMC8723838 34987580

[72] Peng X, Wu X, Wu G, Peng C, Huang B, Huang M, et al. MiR-129-2-3p Inhibits Esophageal Carcinoma Cell Proliferation, Migration, and Invasion via Targeting DNMT3B. Curr Mol Pharmacol. 2023;16:116–23. 10.2174/1874467215666220308122716. 35260066

[73] Lane AN, Fan TW. Regulation of mammalian nucleotide metabolism and biosynthesis. Nucleic Acids Res. 2015;43:2466–85. 10.1093/nar/gkv047. PMC4344498 25628363

[74] Kurmi K, Haigis MC. Nitrogen Metabolism in Cancer and Immunity. Trends Cell Biol. 2020;30:408–24. 10.1016/j.tcb.2020.02.005. PMC7386658 32302552

[75] Zhang Z, Xiahou Z, Wu W, Song Y. Nitrogen Metabolism Disorder Accelerates Occurrence and Development of Lung Adenocarcinoma: A Bioinformatic Analysis and In Vitro Experiments. Front Oncol. 2022;12:916777. 10.3389/fonc.2022.916777. PMC9315097 35903696

[76] Long Z, Zhu T, Zhou Y, Xiang Z, Zeng Q, Qiu Y, et al. The Relationship Between Blood Urea Nitrogen to Creatinine Ratio and Severe Acute Exacerbation of COPD Patients: A Propensity Score Matching Study. Int J Chron Obstruct Pulmon Dis. 2024;19:2529–43. 10.2147/copd.S488394. PMC11606149 39618501

[77] Wu C, Yu H, Liang F, Huang X, Jiang B, Lou Z, et al. Hypoxia inhibits the iMo/cDC2/CD8 + TRMs immune axis in the tumor microenvironment of human esophageal cancer. J Immunother Cancer. 2024;12. 10.1136/jitc-2024-008889. PMC11227851 38964786

[78] Kunkel TA, Erie DA. DNA mismatch repair. Annu Rev Biochem. 2005;74:681–710. 10.1146/annurev.biochem.74.082803.133243. 15952900

[79] Carvalho FM, Carvalho JP. Unraveling the Heterogeneity of Deficiency of Mismatch Repair Proteins in Endometrial Cancer: Predictive Biomarkers and Assessment Challenges. Cancers (Basel). 2024;16. 10.3390/cancers16203452. PMC11505891 39456546

[80] Cai X, Lin W, Wu F, Song G, Qian Z, Wang Y. RARB associated with MSI, affects progression and prognosis of gastric cancer. BMC Gastroenterol. 2024;24:285. 10.1186/s12876-024-03339-z. PMC11342619 39179979

[81] Prasad P, Chongtham J, Tripathi SC, Ganguly NK, Mittal SA, Srivastava T. Targeted inhibition of NRF2 reduces the invasive and metastatic ability of HIP1 depleted lung cancer cells. Biochem Biophys Res Commun. 2024;733:150676. 10.1016/j.bbrc.2024.150676. 39303527

[82] Pupo E, Avanzato D, Middonti E, Bussolino F, Lanzetti L. KRAS-Driven Metabolic Rewiring Reveals Novel Actionable Targets in Cancer. Front Oncol. 2019;9:848. 10.3389/fonc.2019.00848. PMC6730590 31544066

[83] Zhang B, Li J, Hua Q, Wang H, Xu G, Chen J, et al. Tumor CEMIP drives immune evasion of colorectal cancer via MHC-I internalization and degradation. J Immunother Cancer. 2023;11. 10.1136/jitc-2022-005592. PMC9815088 36596591

[84] Chan ET, Kural C. Targeting endocytosis to sensitize cancer cells to programmed cell death. Biochem Soc Trans. 2024;52:1703–13. 10.1042/bst20231332. PMC11519968 39092762

[85] Goyal K, Konar A, Kumar A, Koul V. Bacosides Encapsulated in Lactoferrin Conjugated PEG-PLA-PCL-OH Based Polymersomes Act as Epigenetic Modulator in Chemically Induced Amnesia. Neurochem Res. 2020;45:796–808. 10.1007/s11064-020-02953-z. 31960226

[86] Rosenberg HF, Dyer KD, Foster PS. Eosinophils: changing perspectives in health and disease. Nat Rev Immunol. 2013;13:9–22. 10.1038/nri3341. PMC4357492 23154224

[87] Grisaru-Tal S, Itan M, Klion AD, Munitz A. A new dawn for eosinophils in the tumour microenvironment. Nat Rev Cancer. 2020;20:594–607. 10.1038/s41568-020-0283-9. 32678342

[88] Artham S, Chang CY, McDonnell DP. Eosinophilia in cancer and its regulation by sex hormones. Trends Endocrinol Metab. 2023;34:5–20. 10.1016/j.tem.2022.11.002. PMC10122120 36443206

[89] Spiegel GW, Ashraf M, Brooks JJ. Eosinophils as a marker for invasion in cervical squamous neoplastic lesions. Int J Gynecol Pathol. 2002;21:117–24. 10.1097/00004347-200204000-00003. 11917220

[90] Bishara S, Griffin M, Cargill A, Bali A, Gore ME, Kaye SB, et al. Pre-treatment white blood cell subtypes as prognostic indicators in ovarian cancer. Eur J Obstet Gynecol Reprod Biol. 2008;138:71–5. 10.1016/j.ejogrb.2007.05.012. 17644243

[91] von Wasielewski R, Seth S, Franklin J, Fischer R, Hübner K, Hansmann ML, et al. Tissue eosinophilia correlates strongly with poor prognosis in nodular sclerosing Hodgkin’s disease, allowing for known prognostic factors. Blood. 2000;95:1207–13. 10666192

[92] Lopez-Perez D, Prados-Lopez B, Galvez J, Leon J, Carazo A. Eosinophils in Colorectal Cancer: Emerging Insights into Anti-Tumoral Mechanisms and Clinical Implications. Int J Mol Sci. 2024;25. 10.3390/ijms25116098. PMC11172675 38892286

[93] Jacobse J, Aziz Z, Sun L, Chaparro J, Pilat JM, Kwag A, et al. Eosinophils Exert Antitumorigenic Effects in the Development of Esophageal Squamous Cell Carcinoma. Cell Mol Gastroenterol Hepatol. 2023;16:961–83. 10.1016/j.jcmgh.2023.08.005. PMC10630122 37574015

[94] Kwon HJ, Lee S, Lee HH, Cho H, Jung J. Korean Red Ginseng Enhances Immunotherapeutic Effects of NK Cells via Eosinophils in Metastatic Liver Cancer Model. Nutrients. 2021;14. 10.3390/nu14010134. PMC8747263 35011007

[95] Shelton A, Green RH, Bradding P, Free CM. Peripheral blood eosinophil count correlates with survival in lung cancer. Lung Cancer. 2010;67:S40–1. 10.1016/S0169-5002(10)70123-9.

[96] Yang X, Wang L, Du H, Lin B, Yi J, Wen X, et al. Prognostic impact of eosinophils in peripheral blood and tumor site in patients with esophageal squamous cell carcinoma treated with concurrent chemoradiotherapy. Med (Baltim). 2021;100:e24328. 10.1097/md.0000000000024328. PMC7837956 33546064

[97] Cui K, Hu S, Mei X, Cheng M. Innate Immune Cells in the Esophageal Tumor Microenvironment. Front Immunol. 2021;12:654731. 10.3389/fimmu.2021.654731. PMC8113860 33995371

[98] Fujii SI, Shimizu K. Immune Networks and Therapeutic Targeting of iNKT Cells in Cancer. Trends Immunol. 2019;40:984–97. 10.1016/j.it.2019.09.008. 31676264

[99] Nelson A, Lukacs JD, Johnston B. The Current Landscape of NKT Cell Immunotherapy and the Hills Ahead. Cancers (Basel). 2021;13. 10.3390/cancers13205174. PMC8533824 34680322

[100] Tachibana T, Onodera H, Tsuruyama T, Mori A, Nagayama S, Hiai H, et al. Increased intratumor Valpha24-positive natural killer T cells: a prognostic factor for primary colorectal carcinomas. Clin Cancer Res. 2005;11:7322–7. 10.1158/1078-0432.Ccr-05-0877. 16243803

[101] Lundgren S, Warfvinge CF, Elebro J, Heby M, Nodin B, Krzyzanowska A, et al. The Prognostic Impact of NK/NKT Cell Density in Periampullary Adenocarcinoma Differs by Morphological Type and Adjuvant Treatment. PLoS ONE. 2016;11:e0156497. 10.1371/journal.pone.0156497. PMC4898776 27275582

[102] Metelitsa LS, Wu HW, Wang H, Yang Y, Warsi Z, Asgharzadeh S, et al. Natural killer T cells infiltrate neuroblastomas expressing the chemokine CCL2. J Exp Med. 2004;199:1213–21. 10.1084/jem.20031462. PMC2211904 15123743

[103] Altman JB, Benavides AD, Das R, Bassiri H. Antitumor Responses of Invariant Natural Killer T Cells. J Immunol Res. 2015;2015:652875. 10.1155/2015/652875. PMC4620262 26543874

[104] Yanagisawa K, Seino K, Ishikawa Y, Nozue M, Todoroki T, Fukao K. Impaired proliferative response of V alpha 24 NKT cells from cancer patients against alpha-galactosylceramide. J Immunol. 2002;168:6494–9. 10.4049/jimmunol.168.12.6494. 12055270

[105] Sarvaria A, Madrigal JA, Saudemont A. B cell regulation in cancer and anti-tumor immunity. Cell Mol Immunol. 2017;14:662–74. 10.1038/cmi.2017.35. PMC5549607 28626234

[106] Nielsen JS, Sahota RA, Milne K, Kost SE, Nesslinger NJ, Watson PH, et al. CD20 + tumor-infiltrating lymphocytes have an atypical CD27- memory phenotype and together with CD8 + T cells promote favorable prognosis in ovarian cancer. Clin Cancer Res. 2012;18:3281–92. 10.1158/1078-0432.Ccr-12-0234. 22553348

[107] Al-Shibli KI, Donnem T, Al-Saad S, Persson M, Bremnes RM, Busund LT. Prognostic effect of epithelial and stromal lymphocyte infiltration in non-small cell lung cancer. Clin Cancer Res. 2008;14:5220–7. 10.1158/1078-0432.Ccr-08-0133. 18698040

[108] Nedergaard BS, Ladekarl M, Nyengaard JR, Nielsen K. A comparative study of the cellular immune response in patients with stage IB cervical squamous cell carcinoma. Low numbers of several immune cell subtypes are strongly associated with relapse of disease within 5 years. Gynecol Oncol. 2008;108:106–11. 10.1016/j.ygyno.2007.08.089. 17945335

